# Machine-Learning Prediction of Curie Temperature from Chemical Compositions of Ferromagnetic Materials

**DOI:** 10.1021/acs.jcim.4c00947

**Published:** 2024-08-07

**Authors:** Son Gyo Jung, Guwon Jung, Jacqueline M. Cole

**Affiliations:** † Cavendish Laboratory, Department of Physics, University of Cambridge, J. J. Thomson Avenue, Cambridge, CB3 0HE, U.K.; ‡ ISIS Neutron and Muon Source, STFC Rutherford Appleton Laboratory, Harwell Science and Innovation Campus, Didcot, Oxfordshire OX11 0QX, U.K.; § Research Complex at Harwell, Rutherford Appleton Laboratory, Harwell Science and Innovation Campus, Didcot, Oxfordshire OX11 0FA, U.K.; ∥ Scientific Computing Department, STFC Rutherford Appleton Laboratory, Harwell Science and Innovation Campus, Didcot, Oxfordshire OX11 0QX, U.K.

## Abstract

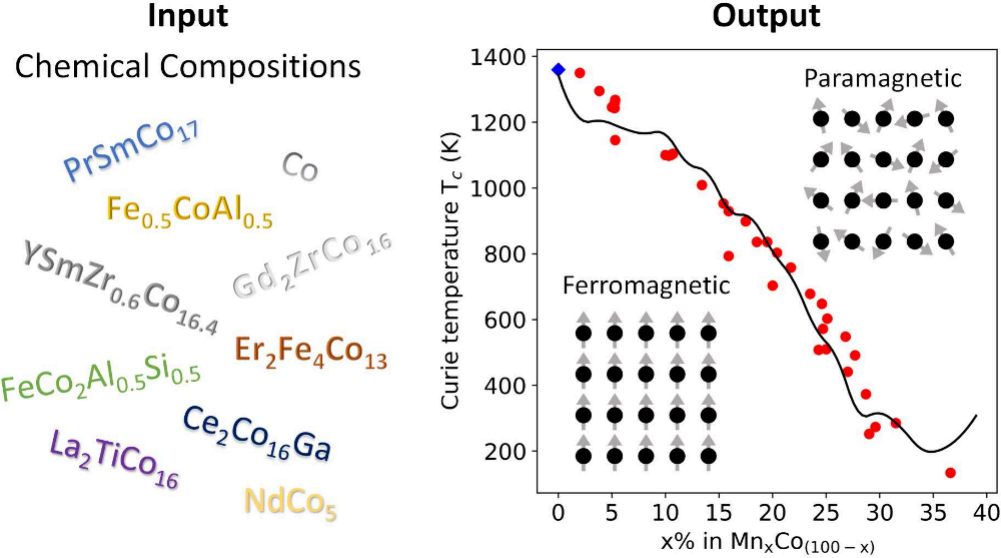

Room-temperature ferromagnets are high-value targets for discovery given the ease by which they could be embedded within magnetic devices. However, the multitude of potential interactions among magnetic ions and their surrounding environments renders the prediction of thermally stable magnetic properties challenging. Therefore, it is vital to explore methods that can effectively screen potential candidates to expedite the discovery of novel ferromagnetic materials within highly intricate feature spaces. To this end, we explore machine-learning (ML) methods as a means to predict the Curie temperature (*T*_*c*_) of ferromagnetic materials by discerning patterns within materials databases. This study emphasizes the importance of feature analysis and selection in ML modeling and demonstrates the efficacy of our gradient-boosted statistical feature-selection workflow for training predictive models. The models are fine-tuned through Bayesian optimization, using features derived solely from the chemical compositions of the materials data, before the model predictions are evaluated against literature values. We have collated ca. 35,000 *T*_*c*_ values and the performance of our workflow is benchmarked against state-of-the-art algorithms, the results of which demonstrate that our methodology is superior to the majority of alternative methods. In a 10-fold cross-validation, our regression model realized an *R*^2^ of (0.92 ± 0.01), an MAE of (40.8 ± 1.9) K, and an RMSE of (80.0 ± 5.0) K. We demonstrate the utility of our ML model through case studies that forecast *T*_*c*_ values for rare-earth intermetallic compounds and generate magnetic phase diagrams for various chemical systems. These case studies highlight the importance of a systematic approach to feature analysis and selection in enhancing both the predictive capability and interpretability of ML models, while being devoid of potential human bias. They demonstrate the advantages of such an approach over a mere reliance on algorithmic complexity and a black-box treatment in ML-based modeling within the domain of computational materials science.

## 1 Introduction

Magnetic materials have played a pivotal role in driving remarkable technological advancements, most notably in the realm of data storage, where their intrinsic physical properties have been exploited in conjunction with semiconductor technology. This has fundamentally transformed methods that are employed to encode and retain data bits, resulting in a technological paradigm shift, an accompanying proliferation of consumer applications in data storage, and the emergence of spin electronics as a novel research field.^1−5^

These advances have stimulated efforts to better understand the underlying physics that governs the responses of magnetic materials to various energy terms and external factors such as temperature.^1,6^ Such responses engender intricate structure–property relationships in magnetic materials. These are characterized by the interactions between magnetic spins, or moments, which are themselves contingent upon both the crystal geometry and chemical composition of the materials.

Moreover, a magnetic material experiences a loss of collective magnetic order at a certain temperature. For ferromagnetic materials, this phase-transition temperature is known as the Curie temperature (*T*_*c*_). Their ordered magnetic properties cease at *T*_*c*_ or above, where only paramagnetic effects are observed; these have limited utility. Therefore, materials with a high *T*_*c*_ values are sought after to attain thermally stable magnetic states or magnetization for functional applications. To this end, ferromagnetic materials whose *T*_*c*_ value is significantly greater than room temperature are particularly attractive. However, the multitude of potential interactions among magnetic ions and their surrounding environments renders the prediction of magnetic behavior and properties challenging.

The exploration of magnetic materials has primarily been steered by experimental research efforts that rely on trial-and-error methods. Such research is very time-intensive, incurs significant operational costs and necessitates a substantial and sustained level of specialist human capital since a rich amount of domain knowledge is critical to research progress. Therefore, the realm of magnetic materials discovery stands to gain from data-driven methodologies that facilitate the targeted design of novel materials based on a desired property, particularly through the application of machine learning (ML).

The accessibility of large volumes of chemical data, coupled with the rise of big-data initiatives, have resulted in a growing interest in data-driven materials discovery. The proficiency of data science in processing and analyzing large-scale, high-dimensional data sets can realize a design-to-device pipeline for materials discovery at a pace unattainable by conventional experimental processes. Data-driven approaches leverage materials informatics and ML.^7,8^ A typical materials-informatics workflow involves transforming chemical data into a machine-readable format using feature descriptors.^9−11^ The generated features are subsequently used for model training, which facilitates the statistical prediction of: (i) properties of unseen chemical materials via a regression analysis or (ii) the specific class or category to which materials are associated using a classification algorithm. The rationale is to empower ML models to deduce relationships between chemical compositions, material structures and their properties, which exceed the capability of manual analysis. These techniques have already demonstrated their prowess in accurately predicting chemical structures and properties for a variety of materials applications.^8,12−16^ This includes the use of multifidelity modeling strategies that harness high-throughput computational calculations and experimental measurements in tandem.^17,18^ These examples showcase the effectiveness of materials screening for the realization of novel materials within highly complex feature spaces.

Various ML techniques have been employed in order to predict *T*_*c*_ values for a range of ferromagnetic material applications. For instance, Court et al.^19^ identified magnetocaloric effects in 2,448 chemical compounds from the Heusler alloy family, whose *T*_*c*_ values were mined from the scientific literature using the natural-language-processing toolkit, ChemDataExtractor.^20,21^ They trained a gradient-boosting regression model using the XGBoost python library^22^ with 58 element-level features and the magnetic field features, realizing a coefficient of determination (*R*^2^) of 0.71 and a mean absolute error (MAE) of 59.8 K. This model was leveraged by Ucar et al.^23^ in their study of magnetic entropy prediction.

Long et al.^24^ conducted a study aimed at advancing the design of ferromagnetic materials using ML techniques. They employed random-forest algorithms for classifying materials as ferromagnetic or antiferromagnetic based on their magnetic ground states and for predicting the *T*_*c*_ value of ferromagnets. The study realized a classification accuracy of 87% and an *R*^2^ of 91% for the regression task. Their random-forest algorithm was trained on a data set of 1749 ferromagnetic intermetallic compounds, intentionally excluding oxides and compounds that lack Cr, Mn, Fe, Co, and Ni atoms. Additionally, the analysis incorporated 139 chemical and 26 structural descriptor features for each compound.

Dam et al.^25^ performed a regression-based feature-selection process to analyze and predict *T*_*c*_ in binary alloy compounds composed of 3*d* transition metals and 4*f* rare-earth elements, using ML techniques. They found that model accuracy was optimized when the first 5 to 10 descriptor features were used, with the concentration of the rare-earth element being the most salient among the 28 descriptors utilized. The study reported an *R*^2^ of 0.96 and an MAE of 41 K, using a limited data set comprised of 108 experimental data. Additionally, they noted a gradual decrease in model performance with the incorporation of additional descriptors, aligning with expectations.

Notably, Nelson et al.^26^ conducted a ML-driven study that predicted *T*_*c*_ values of ferromagnetic materials solely from their chemical composition. They trained a random-forest regression model using features based on their chemical compositions with ca. 2,500 chemical compounds. Their final model was trained using 129 features and achieved an *R*^2^ of 0.87 and an MAE of 57 K on the test set, while a cross-validation *R*^2^ of 0.81 was realized. A similar study was carried out by Belot et al.^27^ in an attempt to predict *T*_*c*_ values using a larger data set of ferromagnetic materials, while examining a diverse range of methods for material representation. They trained and validated a random-forest model and a k-nearest neighbors algorithm using two sets of data, first with ca. 2,500 and second with ca. 3,000 chemical compounds. They reported that the random-forest model realized the highest accuracy using 85 features based on chemical composition, and the use of complex descriptors and dimensionality reduction did not improve the prediction results. In 3-fold cross-validation, an MAE of 73 K with a standard deviation of 3.2 K was achieved using the first data set, while an MAE of 71 K with a standard deviation of 2.3 K was achieved on the combined data sets. Another study using a random-forest regression was conducted by Singh et al.,^28^ which attained a 5-fold cross-validation *R*^2^ of 0.91 with a root-mean-square error (RMSE) of 59 K. Nevertheless, this study used a relatively small data set of 220 ferromagnetic and ferrimagnetic compounds to analyze rare-earth-based materials. Moreover, a linear regression was used by Sanvito et al.^29^ in order to accelerate the discovery of new ferromagnets in the Heusler alloy family. The regression was calibrated on experimental measurements of ca. 60 chemical compounds. A typical error value in the prediction of *T*_*c*_ values was reported to be in the range of 50 K for two chemical classes of the form Co_2_YZ and X_2_MnZ.

This study presents a new way to predict *T*_*c*_ values, to help researchers deliver more accurate and transparent data-driven designs of ferromagnetic materials through a systematic integration of feature engineering, analyses, selection, and optimization processes.^15^ Our proposed workflow, referred to as gradient-boosted statistical feature selection (GBFS) hereafter, integrates a distributed gradient boosting framework, in conjunction with exploratory data and statistical analyses and multicollinearity treatments, to discern a subset of features that is highly relevant to the target variable or class within a complex feature space; this affords minimal feature redundancy and maximal relevance to the target variable or classes.

Our workflow is generalizable, as has already been demonstrated through its use in successfully predicting material-property relationships in other areas of scientific research using data from DFT calculations^15^ and experimental measurements.^17,18^ Here, we apply the GBFS workflow to predict *T*_*c*_ values of ferromagnetic materials using literature values as training data that emanate predominately from experimental measurements. The performance of our models is compared to state-of-the-art ML approaches that predict *T*_*c*_ values. In order to impose a stringent standard on our modeling approach and to ensure a direct comparison with state-of-the-art results, we restrict our descriptor sets exclusively to those derived from chemical compositions, abstaining from complex feature descriptors. This decision recognizes that experimental *T*_*c*_ values reported in the literature often lack comprehensive crystallographic information, while chemical compositions are readily available.

**Figure 1:**
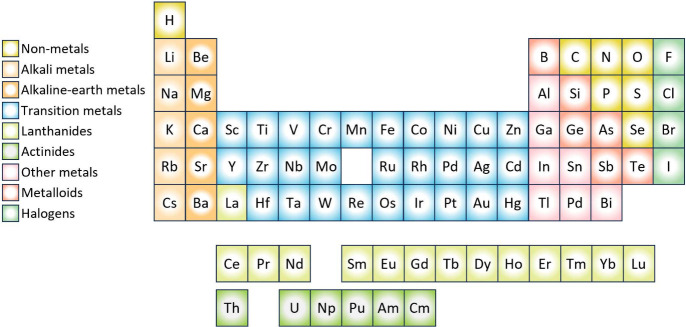
Periodic table highlighting the compositional space represented by both Data sets 1 and 2 that are used in this work.

We herein collate a database comprising ca. 35,000 *T*_*c*_ values from the scientific literature. This database is divided into two segments. The first segment (Data set 1) includes *T*_*c*_ values sourced from a variety of publications,^30−36^ predominantly being experimental values reported by Nelson et al.^26^ and Belot et al.^27^ Data set 1 serves as the foundation for conducting a regression analysis of *T*_*c*_ values, showcasing the effectiveness of our GBFS workflow within this domain. Moreover, it facilitates an equitable comparison of our modeling approach with state-of-the-art models developed using a comparable data set. We broaden our analysis to the second data set (Data set 2) by incorporating *T*_*c*_ values from AtomWork^37^ into a blind-test scenario, exploring potential applications of our ML models beyond a conventional benchmarking evaluation against the state-of-the-art models. By ensuring that there is no overlap of chemical composition with Data set 1, we will show that the blind-test prediction notably identifies 90 chemical compositions that exhibit *T*_*c*_ values which exceed room temperature, a finding that is subsequently corroborated by experimental measurements reported in the scientific literature. Figure 1 depicts the periodic table, highlighting the compositional space covered by these data sets, which exhibit identical chemical coverage.

Furthermore, this study demonstrates the utility of our ML models through two primary applications: (i) the prediction of *T*_*c*_ values for rare-earth intermetallic compounds and (ii) the generation of magnetic phase diagrams of various chemical compounds, where we assess how well our ML models perform when given the task to make out-of-distribution predictions. We emphasis that there exists no overlaps between the previously aforementioned data sets. Our choice of evaluation data sets ensures that each of our ML modeling analyses maintains its distinctiveness, and our results are juxtaposed with corresponding studies utilizing identical or similar data sets for comparative purposes.

Overall, we will show that our GBFS approach highlights the importance of thorough feature analysis and judicious selection over merely complex modeling. Thereby, we will notion that a common off-the-shelf model trained on features selected and engineered by the GBFS workflow can yield results that are comparable or superior to those reported in the literature. The workflow additionally provides insights into feature interactions and their relevance to the target variable. The collated database of *T*_*c*_ values and their corresponding chemical compositions is made available (https://github.com/Songyosk/CurieML) to serve as a resource for researchers engaged in the design of ferromagnetic materials.

## 2 Methods

### 2.1 Featurizers

A high-dimensional feature vector was constructed using a set of composition-based descriptors, while incorporating the composition featurizer modules from Matminer^38^ and Pymatgen.^39^ Additional features were generated by computing statistics over elemental attributes that are specific to each chemical composition. These calculations draw from various data sources, including Magpie,^40^ Pymatgen,^39^ Deml,^41^ and neural-network embeddings of elements that have been created using the Materials Graph Network (MEGNet).^42^

In the field of materials informatics, these tools are instrumental for providing a range of composition-based features that are essential for analyzing and predicting material properties. Matminer, developed by researchers at Lawrence Berkeley National Laboratory, is a comprehensive Python library designed for materials analysis. It provides an array of composition-based features derived from elemental properties, including atomic radius, electronegativity, and ionization energy. Matminer enables the computation of various statistical measures related to these properties across constituent chemical elements. Moreover, it offers features related to the ionic character of material bonds, the oxidation states of elements, and electronic structure attributes such as the valence electron configuration of the elements.

Magpie (Materials-Agnostic Platform for Informatics and Exploration) is engineered to generate a diverse array of features from chemical compositions, optimizing their use in ML models. The platform offers a wide range of features derived from elemental properties, encompassing both physical properties—such as density, heat capacity, and thermal expansion—and electronic properties—including electronegativity, valence electron concentration, and electron affinity. Magpie computes statistical measures of these properties for the constituent chemical elements.

Pymatgen (Python Materials Genomics) is a materials analysis library, offering an extensive suite of tools, designed to manage and analyze materials data. In addition to its utility on structural data, Pymatgen also supports the extraction of valuable composition-based features, and it is integrated with the Materials Project databases.^43,44^ The features generated by Pymatgen include the elemental fraction and atomic fraction of elements within the material, as well as comprehensive statistics on the physical and chemical properties of the constituent elements. These properties encompass melting points, boiling points, thermal conductivity, ionization energies, and other relevant parameters, providing a rich data set for materials characterization and predictive modeling.

In essence, these packages enable the computation of four distinct types of attributes or measures. The first type is stoichiometric attributes, which rely solely on the proportions of elements present within a chemical compound. This category encompasses the number of elements in the compound and various L^*p*^ norms of these proportions or fractions. The second type involves elemental property statistics, including the maximum, minimum, mean, range, mode and mean absolute deviation of various elemental properties. In other words, these statistical measures are calculated by considering the properties of each individual element within a composition. This includes attributes such as the average atomic number, the highest group number on the periodic table, and the standard deviation of Mendeleev numbers among elements in a chemical composition. The third type is electronic structure attributes, which calculate the average fraction of electrons from the *s*, *p*, *d*, and *f* valence shells across constituent elements. The fourth type pertains to ionic compound attributes, assessing the potential to form an ionic compound by assuming constituent chemical elements are in a single oxidation state. It also includes measures of the ionic characteristics of a compound based on electronegativity.

MEGNet, an acronym for MatErials Graph Network, represents a graph neural network model tailored for materials science applications. It uses a graph-based framework to extract material properties from the structural configurations of molecules and crystals. MEGNet models, once trained, can function effectively as feature extractors for new materials, where the embeddings they generate are used as input features for subsequent predictive models. These embeddings effectively capture the chemical periodicity and inherent trends observable within the periodic table. While the individual interpretation of these embeddings can pose challenges, their utility is well-documented, particularly in transfer learning applications. For instance, embeddings derived from a model trained on a comprehensive data set can enhance the predictive capabilities of other models that are trained on more constrained or limited data sets. In our study, we employed MEGNet embeddings to augment elemental characterization, facilitating the application of transfer learning from pretrained models to improve the prediction of *T*_*c*_ values.

### 2.2 Gradient-Boosted Statistical Feature Selection Workflow

**Figure 2:**
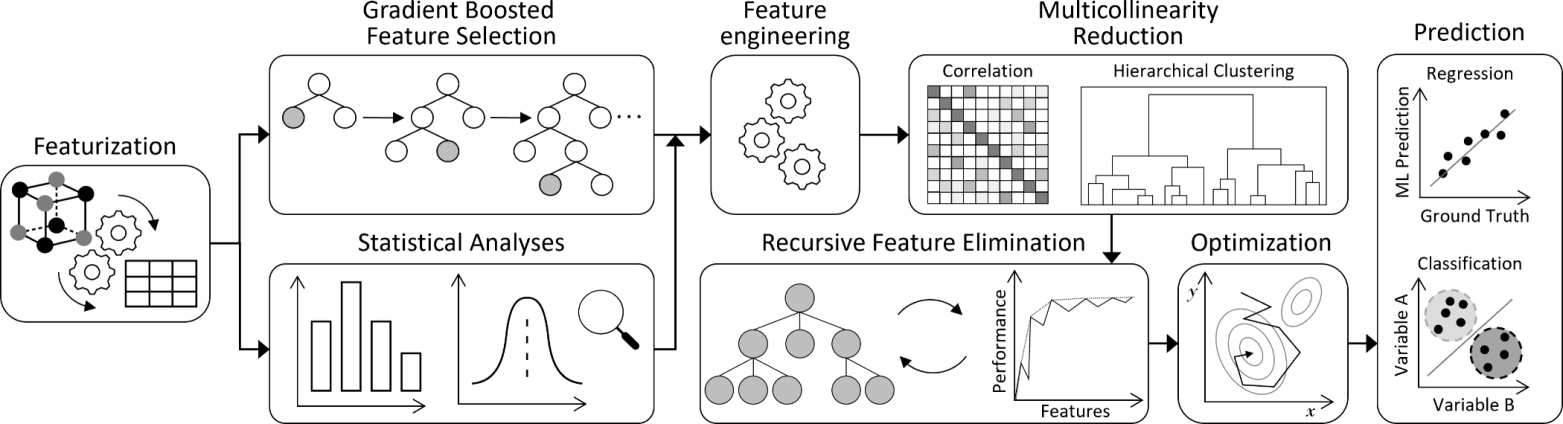
Overview of our operational workflow. Adapted with permission from ref (15). Copyright 2023 the authors. Published by AIP Publishing under a Creative Commons CC BY License.

Figure 2 depicts our overarching GBFS workflow that we applied to predict *T*_*c*_ values solely from chemical composition. Full details of this methodology have been described previously by Jung et al.^17^ The method as applied to this specific *T*_*c*_ prediction challenge is provided herein.

The GBFS workflow integrates several key components: (i) a gradient boosting framework to identify a subset of features that maximize relevance to the target variable or class; (ii) statistical analyses of exploratory features to identify those that are statistically significant to the target variable or class; (iii) a feature engineering step for generating additional features; (iv) a two-step multicollinearity reduction process involving correlation and hierarchical cluster analyses to minimize feature redundancy; (v) a recursive feature-elimination process; and (vi) Bayesian optimization to determine the architecture of the final predictive ML model.

While a comprehensive description of each component of our GBFS workflow has been given by Jung et al.,^15^ we herewith provide a summary of its key attributes to ensure clarity and delineate how our approach differs from the aforementioned studies. Our modeling approach offers significant advantages by being highly systematic and minimizing human intervention during both the feature selection and model development phases. Initially, a comprehensive list of approximately 800 exploratory features is compiled, followed by the computation of the loss reduction attributed to each feature. Concurrently, a suite of statistical tests and analyses based on probability theory and information theory are conducted. These two independent stages effectively identify the most relevant features for the target variable; in this case, the values of *T*_*c*_. These salient features are then used to generate additional features. A default method employed is a brute force approach, which requires no domain knowledge, although one can opt for manual intervention at this stage to guide feature engineering. The most relevant and statistically significant features, along with the newly engineered features, are then evaluated for multicollinearity.

The initial step in addressing multicollinearity involves eliminating highly correlated features based on a predefined correlation threshold. This is followed by hierarchical clustering analysis to group similar features. A linkage threshold is set, allowing the algorithm to automatically select one feature from each cluster to represent that cluster. The underlying rationale is that similar information can be derived from a single representative feature within a given cluster group, thereby streamlining the feature space without loss of critical information. Subsequently, recursive feature elimination is performed, where a greedy-based search method prunes features in a recursive manner until the desired number of features is reached, or no model deterioration is observed. At this stage, permutation importance analysis is also carried out, which involves randomly shuffling the values of a single feature to observe the impact on performance metrics.

This meticulous process leads to a refined subset of features that are used to perform Bayesian optimization. In this study, for instance, 36 features were systematically selected from an initial set of approximately 800 features. The optimization stage autonomously identifies the most effective model architecture using solely the training set, without the need for human intervention throughout the process. Once the final predictive model has been optimized, it is evaluated using the test set—marking the first and only time that this data set is used. This rigorous approach ensures that our model is both robust and effective, leveraging systematic methodologies to enhance predictive accuracy and reliability, while eliminating potential human bias.

This comprehensive strategy ensures that the selected features contribute optimally to the predictive accuracy of our model, while effectively minimizing the effects of high correlations and redundancy among the input features. By significantly reducing the complexity of the feature space, this approach enables one to address potential overfitting issues and inherently performs regularization to achieve model generalization. This underscores the advanced and reliable nature of our highly systematic analytical approach. In the following sections, we present the results that are associated with each component of the GBFS workflow.

For the regression analysis, we computed the mean absolute error (MAE), the mean squared error (MSE) and the coefficient of determination that is defined as the square of the Pearson correlation coefficient, *R*, according to
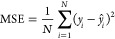
1
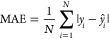
2
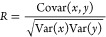
3where *y* and *ŷ* are the true and predicted values, respectively, over a number of samples, *N*;  is the covariance between *x* and *y*;  and  are the variance of *x* and *y*, respectively; and  and  are the mean of *x* and *y*, respectively. The range of *R* is [−1, 1] and its value indicates the extent by which a quantity has a linear tendency to change as the values of another are varied.

## 3 Results and Discussion

### 3.1 Regression Analysis of Curie Temperatures

#### 3.1.1 Overall Performance Evaluation

We present the results of the regression analysis of *T*_*c*_ values that employed Data set 1, which encompasses ca. 11,000 chemical compounds, among which ca. 7,200 represent distinct chemical compositions. The data set was segmented according to a train-to-test split ratio of 4:1 through random splitting. For duplicate chemical compositions, the median values of *T*_*c*_ were calculated. The decision to adopt the median as the measure of central tendency was motivated by its reduced susceptibility to outliers relative to the mean. The regression was performed by a Bayesian-optimized gradient boosting algorithm using 36 features, which were derived exclusively from their chemical compositions. The selection of these features was made from a pool of more than 800 total features through our GBFS workflow.

**Figure 3:**
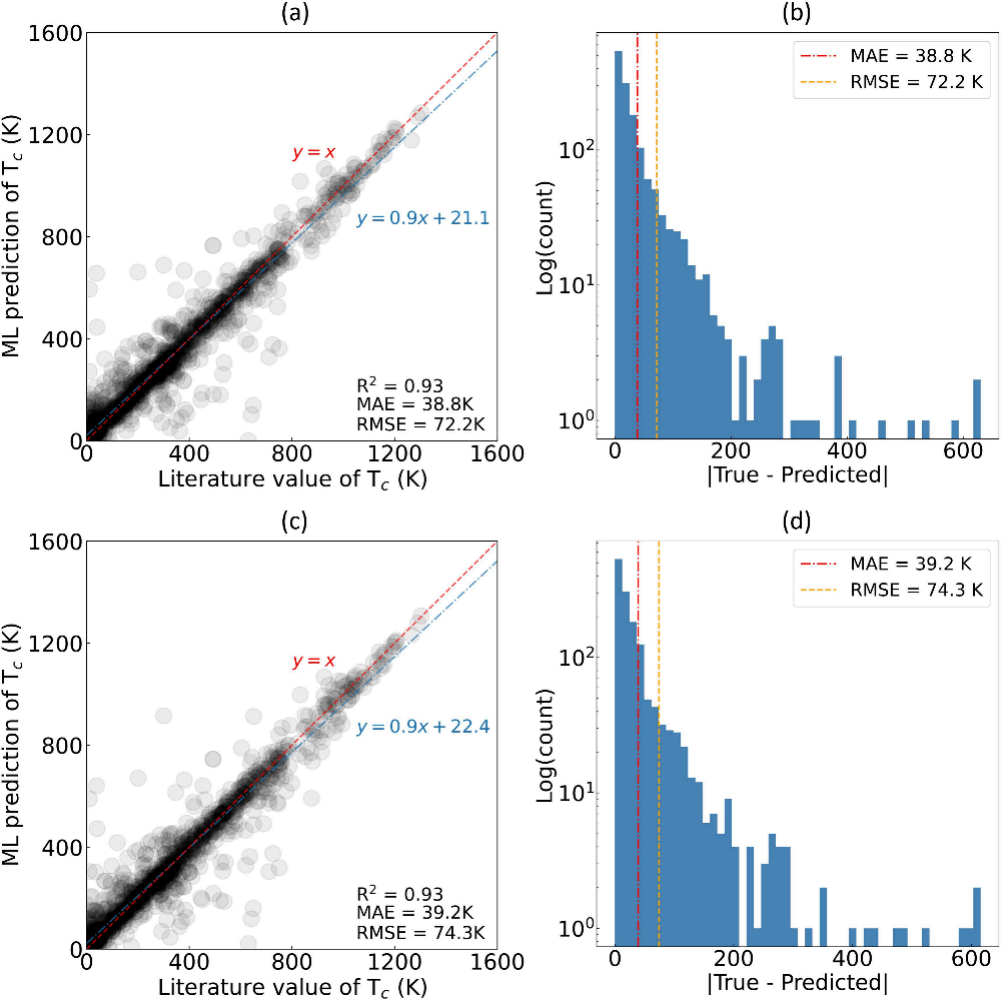
Test set results of the ML-based predictions of *T*_*c*_ against the ground truth for the regression model that has been trained and Bayesian-optimized on the final subset of features selected by our GBFS workflow, where (a) excludes and (c) includes the MEGNet embeddings. The dashed red line is drawn to represent the hypothetical case, where our ML-based predictions would equal the literature values (ground truth). The blue dot-dash line is a linear fit generated using the OLS method. (b) and (d) show the corresponding distribution of absolute errors for (a) and (c), respectively, where the dashed line (−·) in red indicates the MAE and the dashed line (−−) in orange indicates the RMSE.

Figure 3 shows the resulting model performance and error distribution on the test set. The blue dot-dash line represents the line of best fit, established through the Ordinary Least Squares (OLS) method; it depicts the relationship between *T*_*c*_ values reported in the literature and our ML-based predictions. Two distinct regression analyses were conducted: the first without the inclusion of MEGNet embeddings (as shown in Figure 3 (a)) and the second incorporating these embeddings (depicted in Figure 3 (c)). This methodological differentiation arises from the challenges associated with interpreting the physical contributions of the embeddings to the ultimate predictions. Consequently, this strategy enables a comparative examination of how feature interactions influence the prediction of *T*_*c*_ values.

In Figure 3 (a), the linear fit exhibits a gradient of 0.9 and a *y*-intercept of 21.1 K, rounded to one decimal place. The *y*-intercept suggests a minor systematic bias for lower values of *T*_*c*_, while the gradient demonstrates a nearly perfect alignment of our predictions to the literature data, with a small underestimation at large temperatures within the range considered herein. The high efficacy of this linear fit is corroborated by an *R*^2^ value of 0.93, which signifies a high correlation between predictions and the ground truth. Moreover, MAE and RMSE values of 38.8 and 72.2 K were realized, respectively. The higher RMSE is attributable to the increased penalization of predictions that deviate significantly from their true values. The distribution of absolute errors is depicted in Figure 3 (b). A 10-fold cross-validation process was undertaken, affording an *R*^2^ of (0.92 ± 0.01), an MAE of (40.8 ± 1.9) K, and an RMSE of (80.0 ± 5.0) K. Observations indicate comparable performance upon integrating MEGNet embeddings, as illustrated in Figure 3 (c) and the corresponding error distribution in (d). This underscores their effectiveness in facilitating transfer learning.

Notably, our modeling results outperformed the state-of-the-art report by Nelson et al.^26^ (test set *R*^2^ of 0.87 and MAE of 57 K with 129 features and a cross-validation *R*^2^ of 0.81) and Belot et al.^27^ (a cross-validation MAE of 71 K with a standard deviation of 2.3 K with 85 features). These observations can be attributed to two primary factors. First, our study involved the aggregation and analysis of an extensive data set, constituting the largest compilation of *T*_*c*_ data available in the literature. The substantial size of our data set enhances our capacity to discern statistically significant relationships between exploratory features and the target variable. Second, our implementation of the GBFS workflow allowed us to minimize feature redundancy and maximize feature relevance to the target variable. The efficiency of our modeling strategy enabled us to surpass the performance of other studies by employing a significantly lower number of input features, and notably, without resorting to the use of regularization techniques during model optimization. The results on both the test set and the cross-validation process further affirm the generalizability of our model, without overfitting to the training set.

**Figure 4:**
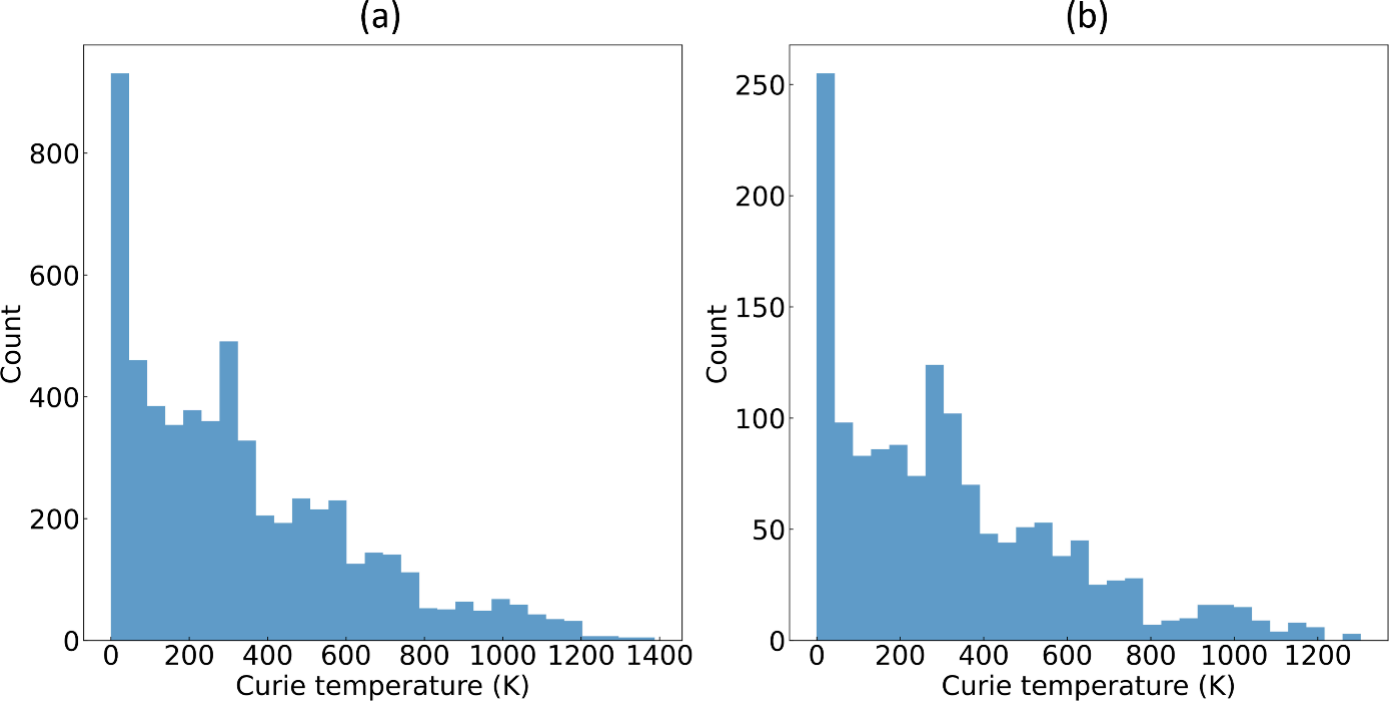
Distribution of Curie temperature (*T*_*c*_) values in (a) the training set and (b) the test set, sourced from Data set 1.

Our model performs with particularly high efficacy where *T*_*c*_ ≲ 500, which stands to reason given the predominant concentration of data within this temperature range. However, the number of data points diminishes rapidly, with increasing temperature, with the highest *T*_*c*_ of 1,388 K being recorded for the elemental metal, cobalt. This diminishing data density at higher temperatures contributes to the observed discrepancies between the ML predictions and the ground truth in this temperature range, as depicted by the lines of best fit (blue) in Figures 3 (a) and (c), which sit below the diagonal lines in red. The population distribution plots for both the training and test sets are illustrated in Figure 4. Nevertheless, our ML modeling approach has exhibited precise predictions of *T*_*c*_ despite the absence of any 3-D structural information. Moreover, our approach does not involve specific treatments to accommodate different crystal forms of the same chemical compound, a phenomenon known as polymorphism. Instead, it relies on capturing the median values of *T*_*c*_ for each chemical composition. Our approach strictly trains the model to be agnostic to crystalline polymorphs. Turning our attention back to the error distribution plots in Figures 3 (b) and (d), which have a logarithmic scale on the *y*-axis, it is evident that the majority of the predictions exhibit errors below ca. 60 K. Upon closer inspection of the chemical compositions in the test set, ca. 82% have an absolute error below 60 K, ca. 65% have an absolute error below 30 K, and ca. 32% have an absolute error below 10 K.

**Figure 5:**
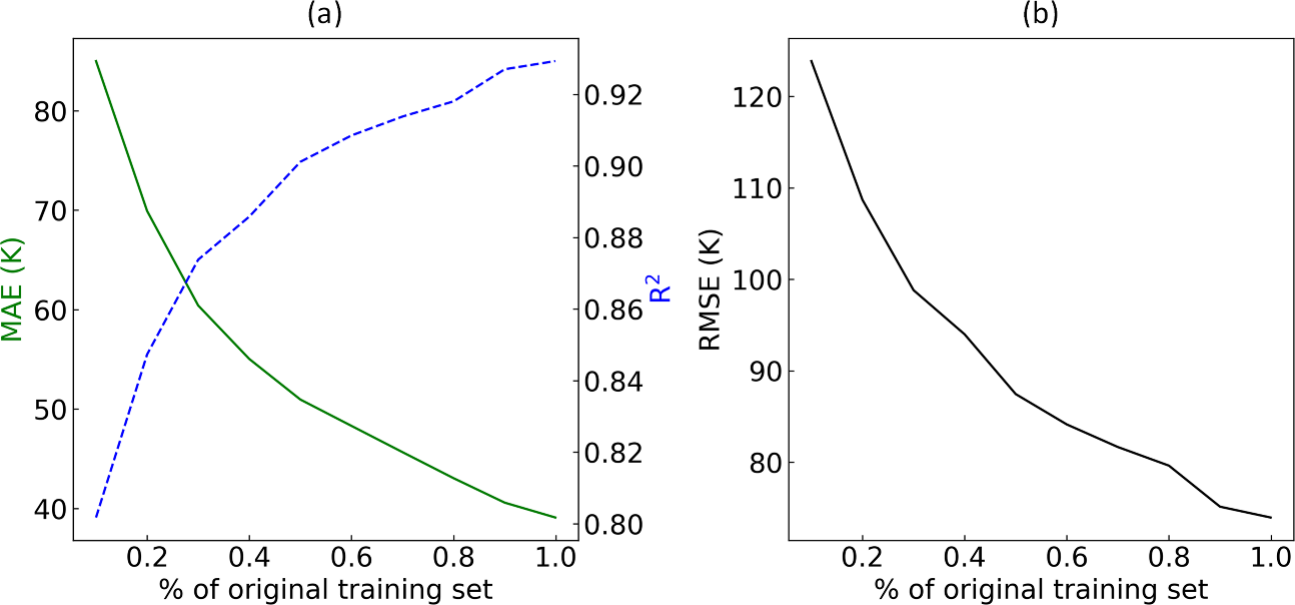
Evaluation of MAE, RMSE, and *R*^2^ conducted by training our ML model on increasingly larger subsets of the original training set, where the data were randomly sampled.

We recognize the importance of assessing how model performance varies with data set size. This evaluation helps to ascertain whether the expanded data set used in this study offers any benefit to the prediction accuracy or if the benefits have plateaued which would indicate no significant advantage in using such a data set. Our analysis evaluated the MAE, RMSE, and *R*^2^ values for increasingly larger subsets of the original training set, where the data were randomly sampled. The results shown in Figure 5 reveal a continuous improvement as the training set size increases, yet without a definitive convergence in these metrics. Therefore, the data set curated for this study not only enhances model performance but it may also hold potential to offer valuable insights to the wider research community.

It is also essential to clarify that our modeling rationale does not overlook the significant impact that structural information can have in enhancing the prediction accuracy. Indeed, the integration of such data would undoubtedly improve our ML model by introducing additional variables that are critical to the predictive outcomes. This is because understanding the arrangement of atoms within a crystal lattice provides insights into the interactions of magnetic moments. For example, body-centered cubic structures, such as those observed in α-iron or ferrite, facilitate specific types of magnetic interactions that are conducive to higher Curie temperatures. Nevertheless, our decision to restrict the feature space exclusively to variables derived from chemical compositions is grounded in two key considerations. First, there is a substantial scarcity of accessible 3-D crystallographic information and it is cost-prohibitive to aggregate such information, which impedes the inclusion of structural data. Second, constraining the feature set in this manner streamlines the predictive modeling process. The inclusion of structural information would necessitate the exclusion of a substantial portion of the data used in this study due to its unavailability. Additionally, should future predictions be made, the featurization stage, which involves generating structural features, would be constrained by the limited availability of necessary crystallographic data, thereby limiting the practical utility of the ML model.

#### 3.1.2 Feature Interpretation

Table 1 summarizes some of the top 10 salient features that contributed to the aforementioned outcomes in our regression analysis. We now seek to rationalize their significant role. The most influential feature, as denoted by the realized total loss reduction, pertains to the mean ground-state magnetic moment of elemental solids for atoms within the chemical composition. This feature is categorized under elemental property statistics, as detailed in Section 2.1. The second most influential feature is the presence of cobalt in the chemical composition. Additional features exhibiting significant relevance with the target variable encompass a range of statistical measures pertaining to the number of *d*-valence electrons or vacant *d*-valence orbitals, the volume of the elemental solid, the periodic table’s group number, electronegativity, instances where the element with the lowest energy molecular orbital (LUMO) is iron, the inclusion of manganese within the composition, the coefficient of linear thermal expansion, thermal conductivity, atomic radius, and the energy of the highest occupied molecular orbital (HOMO).

**Table 1:** A List of Features Identified to Have the Most Relevance in the Prediction of *T*_*c*_ Values

No.	Feature Description
1	Ground-state magnetic moment of elemental solids for atoms within a given chemical composition
2	Presence of cobalt
3	Number of valence electrons in the *d*-orbitals of elements within a given chemical composition
4	Volume of elemental solids for atoms within a given chemical composition
5	Periodic table column number
6	Electronegativity
7	Number of unfilled electrons in the *d*-orbitals of the elements within a given chemical composition
8	HOMO and LUMO energies and their associated chemical elements
9	Presence of manganese
10	MEGNet embeddings

The feature selected with the highest relevance was the mean ground-state magnetic moment of elemental solids for atoms within the chemical composition; this was anticipated, considering its direct material association with the measure of magnetic strength or the tendency of a magnetic moment to align with a magnetic field. The selection of the ground-state magnetic moment as a key feature, while seemingly intuitive, is substantiated by both theoretical and empirical rationales. The ground-state magnetic moment is a fundamental property that defines the magnetic behavior of a material at absolute zero, where the material is in its lowest energy state. Specifically, it quantifies the total magnetic dipole moment in the most stable configuration of the material. This quantification reflects the nature and intensity of the magnetic spins within the material, which are crucial for determining its ferromagnetic characteristics. Thus, the mean ground-state magnetic moment feature is directly linked to *T*_*c*_, as it embodies the strength of the magnetic interactions within the material. A higher magnetic moment typically indicates stronger magnetic interactions, which are pivotal because they dictate the temperature at which thermal energy disrupts the magnetic ordering, thereby influencing *T*_*c*_.

Furthermore, the magnitude and presence of the ground-state magnetic moment act as markers of ferromagnetic properties. Ferromagnetic materials, distinguished by a nonzero ground-state magnetic moment, manifest spontaneous magnetic order below *T*_*c*_ due to the alignment of magnetic moments. Therefore, quantifying these moments offers a direct measure of the extent and nature of ferromagnetism in the material, positioning the mean ground-state magnetic moment as a critical metric for *T*_*c*_.

Empirical research consistently demonstrates a correlation between *T*_*c*_ and the nature and strength of magnetic interactions, as encapsulated by the magnetic moment. This relationship is exploited in predictive modeling, wherein the ground-state magnetic moment serves as a foundational component for estimating *T*_*c*_. For instance, an empirical study conducted by Fecher et al.^45^ has observed that *T*_*c*_ values of Co_2_-based Heusler compounds display a discernible linear correlation with the magnetic moment. Fecher et al. extrapolated such a linear pattern to ascertain that *T*_*c*_ can surpass 1,000 K in Co_2_-based Heusler compounds characterized by a magnetic moment of 6 μ_*B*_ and 30 valence electrons per unit cell (e.g., Co_2_FeSi). This observation additionally underscores the dependence of *T*_*c*_ on both the number of valence electrons and the presence of elemental cobalt in the chemical composition (cf. the second and third-ranked selected features). More broadly, the relevance of such a feature generally applies to alloys that adhere to the Slater-Pauling curve.^46,47^ It is a well-established fact that the magnetization of 3*d* transition metal substitutional alloys, as a function of the valence electron number per atom, forms the Slater-Pauling curve. Similarly, *T*_*c*_ values of these alloys display a systematic pattern relative to the number of valence electrons, a phenomenon that has also been substantiated through first-principles calculations.^48^ Therefore, it is evident that the magnetic moment is correlated with the Slater-Pauling curve, which is discussed in more detail later in this section.

The prominence of cobalt-based attributes as the second most salient feature in predicting *T*_*c*_ aligns with expectations. As previously mentioned, cobalt as an elemental metal exhibits the highest recorded *T*_*c*_ of 1,388 K. Materials with high *T*_*c*_ values predominantly feature cobalt within their chemical composition. As we will see later, subsequent blind-test analysis further reveals that the majority of chemical compositions with *T*_*c*_ ≳ 600 K include both cobalt and iron, indicating the significance of cobalt in achieving elevated *T*_*c*_ values.

The third most salient type of feature is associated with *d*-valence electrons or orbitals, for which a clear rationale exists. Slater^46^ and Pauling^47^ established that the magnetic moments of 3*d* elements and their binary alloys could be characterized by the mean number of valence electrons per atom, offering a simple explanation of the relationship between the number of valence electrons and magnetic moment in ferromagnetic alloys. Specifically, Co_2_-based Heusler compounds adhere to the Slater-Pauling rule, which predicts that the total magnetic moment scales linearly with the number of valence electrons.^45,49,50^ Co_2_-based compounds are situated on the localized part of the Slater-Pauling curve, which is indicative of an increasing magnetic moment with an increasing number of valence electrons.^51^ It is also established that in quaternary half-metallic ferromagnetic materials, the incorporation of a transition metal with 4*d* electrons in conjunction with iron or manganese can result in an elevation of the *T*_*c*_ value. The variation of *T*_*c*_ as a function of the number of valence electrons can be understood through the interatomic exchange interaction parameter.^52^ These findings elucidate one of several interactions among diverse features as discerned through the GBFS workflow. It accentuates the predictive significance of valence electron characteristics in ascertaining magnetic properties, thereby underscoring the intricacy of feature interrelations within the predictive model.

The GBFS workflow identified additional chemical composition-based features that are also of significance. For instance, the average deviation in the periodic table column positions among elements within the chemical composition demonstrates a notable correlation of ca. −0.45 with the target variable, *T*_*c*_. This negative correlation indicates that materials with smaller average deviations in the periodic table column positions among their constituent elements tend to be associated with higher *T*_*c*_ values. This characteristic is prevalent in materials comprising metals such as iron, nickel, cobalt, and rare-earth metals. Likewise, characteristics associated with the periodic table’s group number, atomic radius, and Mendeleev number emerge as salient features identified by the GBFS workflow. These attributes are closely tied to an element’s specific location within the periodic table, underscoring their relevance in the predictive analysis.

Additionally, the inclusion of manganese within the chemical composition demonstrates significant pertinence. While elemental manganese, a metal, does not exhibit ferromagnetism in its pure form, manganese alloys can exist in the form of a Heusler crystal structure, wherein manganese possesses a magnetic moment. More generally, the marked sensitivity of *T*_*c*_ values that is observed in Mn-containing magnetic alloys is well-documented and can be explained via the empirical Castelliz–Kanomata curves.^53,54^ These curves, which have been validated across various Heusler alloys,^55,56^ highlight that the magnetic interactions in Mn depend on the structural parameters, such as the Mn–Mn nearest neighbor distance. Furthermore, there is substantial empirical evidence demonstrating that pressure, and hence the volume, significantly influences *T*_*c*_ values. These findings suggest that the identification of certain features in our ML model can assist in distinguishing materials with high *T*_*c*_ values or materials that deviate from the elementary framework of ferromagnetism.

Another noteworthy feature is the mean volume of the elemental solid among the elements in the chemical composition. The exchange energy (*E*_*ex*_) is a continuum description of the quantum mechanical exchange interaction and is given by
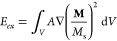
4where *A* is the material-dependent exchange stiffness, **M** is the magnetization, *M*_*s*_ is the saturation magnetization, and the integral is over the volume of the sample.^1^Equation 4 shows that the exchange energy is explicitly linked to the integral over the volume of the sample. This provides a lucid and simple illustration of how volume and shape may affect the exchange energy and coupling and, consequently, influence the prediction of a *T*_*c*_ value.

The final category of features to be addressed involves band gap-related attributes, such as the energies of the HOMO and LUMO or their associated chemical elements, which necessitates further explanation in the context of predicting *T*_*c*_. For instance, the HOMO signifies the highest energy level that contains electrons, analogous to the valence band in Band theory, and it is directly associated with band gap energy. There are several possible theories that could explain the relationship between these features and *T*_*c*_. For example, the influence of the band gap on *T*_*c*_ has been extensively studied by Coey et al.^57^ and Pan et al.^58^ In particular, the *T*_*c*_ values of diluted magnetic oxides (DMOs) are quantitatively explained, based on a bound magnetic polaron mechanism, via the equation
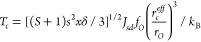
5where *S* is the localized core spin, *s* is the donor electron spin, *x* and δ are the concentrations of magnetic cations and donors, *J*_*sd*_ is the *s*–*d* exchange parameter (which is related to band gap), *f*_*O*_ is the oxygen packing fraction for the oxide, *r*_*c*_^*eff*^ is the effective cation radius, *r*_*O*_ is the radius for the oxide, and *k*_*B*_ is Bolzmann’s constant.^57,58^ The donors form bound magnetic polarons, leading to coupling among the 3*d* moments of the ions within their orbits. When the radius of its orbital is sufficiently large, the ferromagnetic exchange coupling is established from overlap between a hydrogenic electron and the cations within its orbit. This interaction is influenced by the parameter *J*_*sd*_. Equation 5 illustrates the dependency of *T*_*c*_ on band gap via the *J*_*sd*_ parameter, in addition to other crucial factors such as doping and donor concentrations. Indeed, the results of Pan et al.^58^ demonstrate that one can enhance *T*_*c*_ values in DMO materials by manipulating their band gap. This exemplification helps to rationalize why the average number of unfilled *d* orbitals among elements in the chemical composition is also an important feature. Furthermore, it reinforces the identification of *d*-valence electrons as among the most pertinent features for predicting *T*_*c*_, thereby offering additional validation for their relevance.

It is essential to acknowledge that, while the band gap-related features have a relatively modest level of significance compared to other features discussed in this section, their presence is not negligible. Chemical compositions containing oxygen account for approximately 13% of the data set that was used to generate the results shown in Figure 3. Although they represent a minority, these features appear to be helpful in distinguishing between metal oxides and purely metal-based compositions. The ability to identify specific features that differentiate minority classes is of importance. The recognition of such attributes enhances the model’s capability to distinguish between different classes of chemical materials. Moreover, the impact of these features on reducing the loss function during model training further supports their relevance.

The HOMO energy also exhibits a degree of correlation with other selected features, such as the average deviation of electronegativity among elements in the chemical composition, and various features derived from *p*-valence electrons or *p*-orbitals. Such correlations are particularly pronounced for chemical elements that belong to groups 13–18 of the periodic table, notably within the *p*-block. This region of the table is characterized by elements that possess high electronegativity values. The pairing of a metal (characterized by low electronegativity) with a nonmetal element leads to a large difference in orbital energy, a phenomenon that is accentuated as the electronegativity gap between the paired elements widens. It is therefore not surprising that numerous ionic compounds have been identified as being ferromagnetic, exhibiting conductivity levels that are typical of semiconductors. This category of materials encompasses chalcogenides, and halides, or combinations of these groups of the periodic table. In such materials, ions like chromium and europium contribute to the formation of permanent dipole moments. Notably, many rare-earth metals within the lanthanide series exhibit spontaneous magnetization below specific temperatures. Moreover, ferromagnetic ordering is often observed in ionic compounds that feature the spinel crystal structure, which is typified by metal oxides with the general composition AB_2_O_4_.

The final feature to be discussed refers to the MEGNet element embeddings. Upon integrating the MEGNet embeddings, it was noted that 6 of the top 20 most salient features were substituted by these MEGNet embeddings. These learned element embeddings on graph-neutral-network models encode chemical trends in the periodic table. While interpreting individual embeddings can be challenging, previous studies have demonstrated their utility in transfer learning.^15,17,42^ Specifically, these embeddings can be transfer-learned from a material-property model that has been trained on a larger data set to enhance property models with smaller data sets. In this study, we leveraged the acquired embeddings to enhance the predictions of *T*_*c*_ values for ferromagnetic materials.

#### 3.1.3 Gradient Boosted Feature Selection

**Figure 6:**
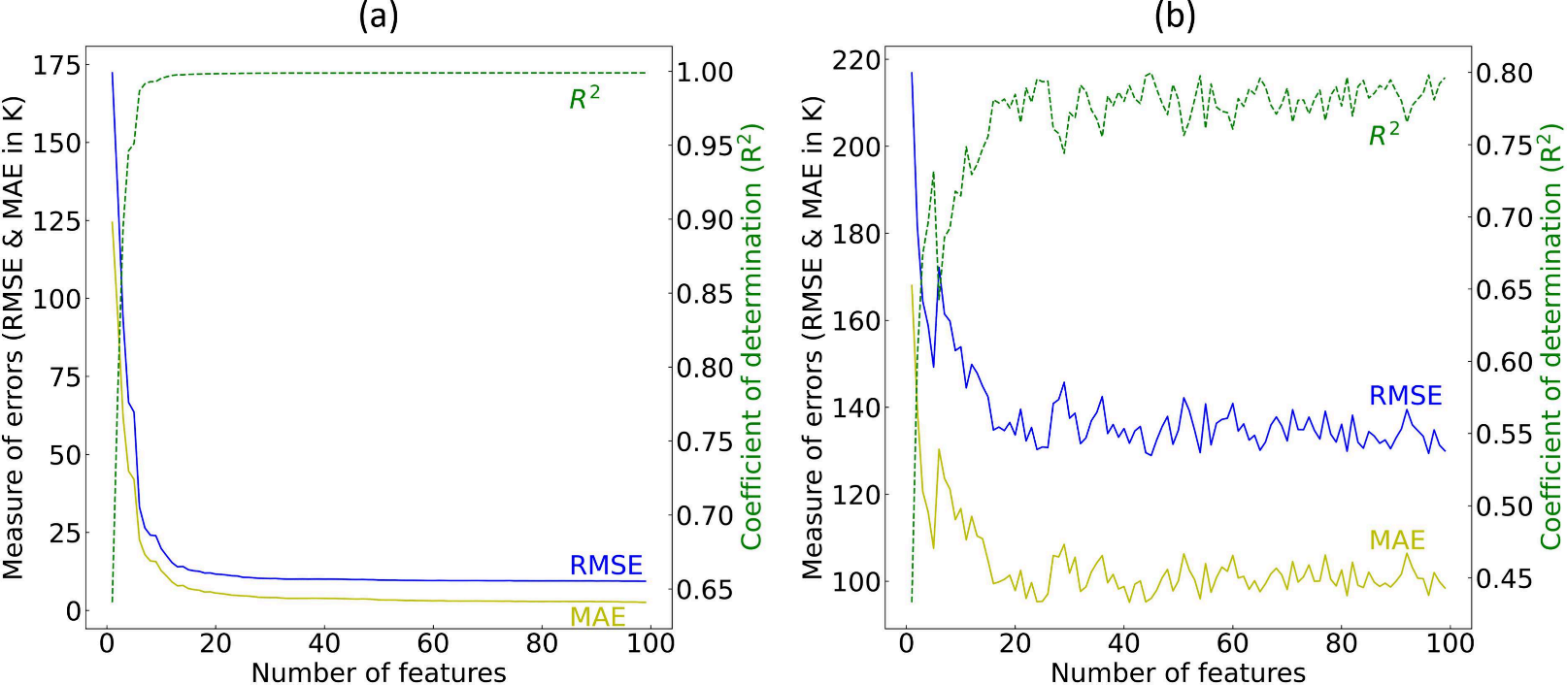
Gradient boosting feature selection results of the regression analysis of *T*_*c*_ value prediction. Model performance of GBDTs on (a) the training set and (b) the validation set, where regression models were trained recursively with an increasing subset of features, beginning from the most relevant feature based on the realized total loss reduction.

Our GBFS workflow selected a final subset of 36 features from over 800 exploratory features. Here, we present the results associated with this selection process. Initially, gradient boosting decision trees (GBDTs) were recursively trained on an increasing subset of features until *R*^2^, MAE, and RMSE converged, whereby the features were originally ranked based on the total loss reduction achieved during the training process. The evolution of these performance metrics over the course of this selection process is illustrated in Figure 6. Evaluation results are shown for both the training set and the validation set. The performance metrics for both sets reached a plateau before ca. 40 features had been included. A comparatively lower performance on the out-of-sample validation set was observed, as expected. Additionally, we noticed a sudden decline in the model performance on the validation set beyond the inclusion of the fifth feature, followed by a subsequent recovery in performance. This phenomenon is not unusual, given that we did not account for multicollinearity at this stage of the workflow, even though it may be present among the exploratory features. In the presence of multicollinearity effects, the total loss reduction is distributed evenly across correlated features, thereby masking the true relevance of individual features to the target variable; see Section 3.1.5 for an explicit consideration of its effects.

#### 3.1.4 Feature Analysis and Feature Engineering

We sought to understand the causal relationship between an exploratory feature and the target variable by concurrently employing hypothesis-based testing methods of a bivariate form. For instance, a comparison of means was conducted using the *F*-test in a one-way analysis of variance (ANOVA). This involves a correlation analysis using *R* for two continuous features, where the ANOVA approach to regression analysis involves converting *R* into a regression *F*-statistic. These hypothesis-based testing methods were employed for statistical inference, with the statistical significance of an exploratory feature being inferred from the test statistics that were generated by testing the hypotheses that pertain to the existence of an association between two features. Additionally, mutual information (MI) analysis was performed. The concept of MI was employed to quantify the level of dependency between two features, measuring the amount of information, or entropy, gained for a feature through the observation of another. For a pair of features, MI assesses the disparity between their joint distribution and the product of their marginal distributions, with a higher MI value indicating a greater dependency between the two features. We adopted an MI estimator based on entropy estimations that are derived from k-nearest neighbor distances.

When assessing the linear association of each continuous exploratory feature with the target variable through a normalized *F*-statistic for relative comparison, we identified that the feature demonstrating the highest linear association with the target variable was the mean ground-state magnetic moment of elemental solids for atoms within a given chemical composition (estimated using Magpie data). Following closely was the fraction of transition metals and the mode of magnetic moment of elemental solids for atoms within a given chemical composition (also estimated using Magpie data), with normalized *F*-statistics reaching 0.75 and 0.68, respectively. Other features exhibited normalized *F*-statistics below ca. 0.6, although these included some with notable loss reduction such as the presence or fractional abundance of cobalt in a chemical composition, the mean volume of elemental solids, the periodic table column or group number of the constituent elements, and the number of *d*-valence electrons.

In parallel, the MI analysis indicated that the greatest amount of entropy gain was realized when considering the mean ground-state magnetic moment of elemental solids for atoms within a given chemical composition. Other notable features with a normalized MI score above 0.8 include statistical measures associated with the fraction of transition metals, the periodic table column or group number of the constituent elements, the number of *d*-valence electrons or unfilled *d*-orbitals, electronegativity, and the Mendeleev number. Since the MI analysis incorporates the k-nearest neighbors method, these results essentially suggest that more accurate predictions of *T*_*c*_ can be achieved by considering statistical measures that pertain to the ground-state magnetic moment of elemental solids and the transition metals (i.e., *d*-block elements). It is noteworthy that the estimation of MI involves assessing the probability-density distribution and marginal distributions of the two variables of interest. However, estimations of these distributions become increasingly challenging in higher-dimensional data, given the limited number of samples with respect to the number of dimensions. This limitation often leads to substantial variations in probability; as a result, the estimated information gain in MI analysis may suffer from the high-dimensionality nature of the data set or an inadequate sample density with respect to the dimension of the feature space. The features identified through the GBFS workflow and statistical analyses were used to engineer new features via the brute-force method. This process resulted in an additional 56 features, leading to a total number of 116 features that formed the preliminary subset of features for the regression analysis.

#### 3.1.5 Multicollinearity Reduction, Permutation Analysis, and Recursive Feature Elimination

In the next phase of the GBFS workflow, we address multicollinearity reduction within the data set, assess the permutation importance of the selected features, and conduct recursive feature elimination to ascertain the final subset of features that will go forward for Bayesian optimization of the final predictive ML model.

**Figure 7:**
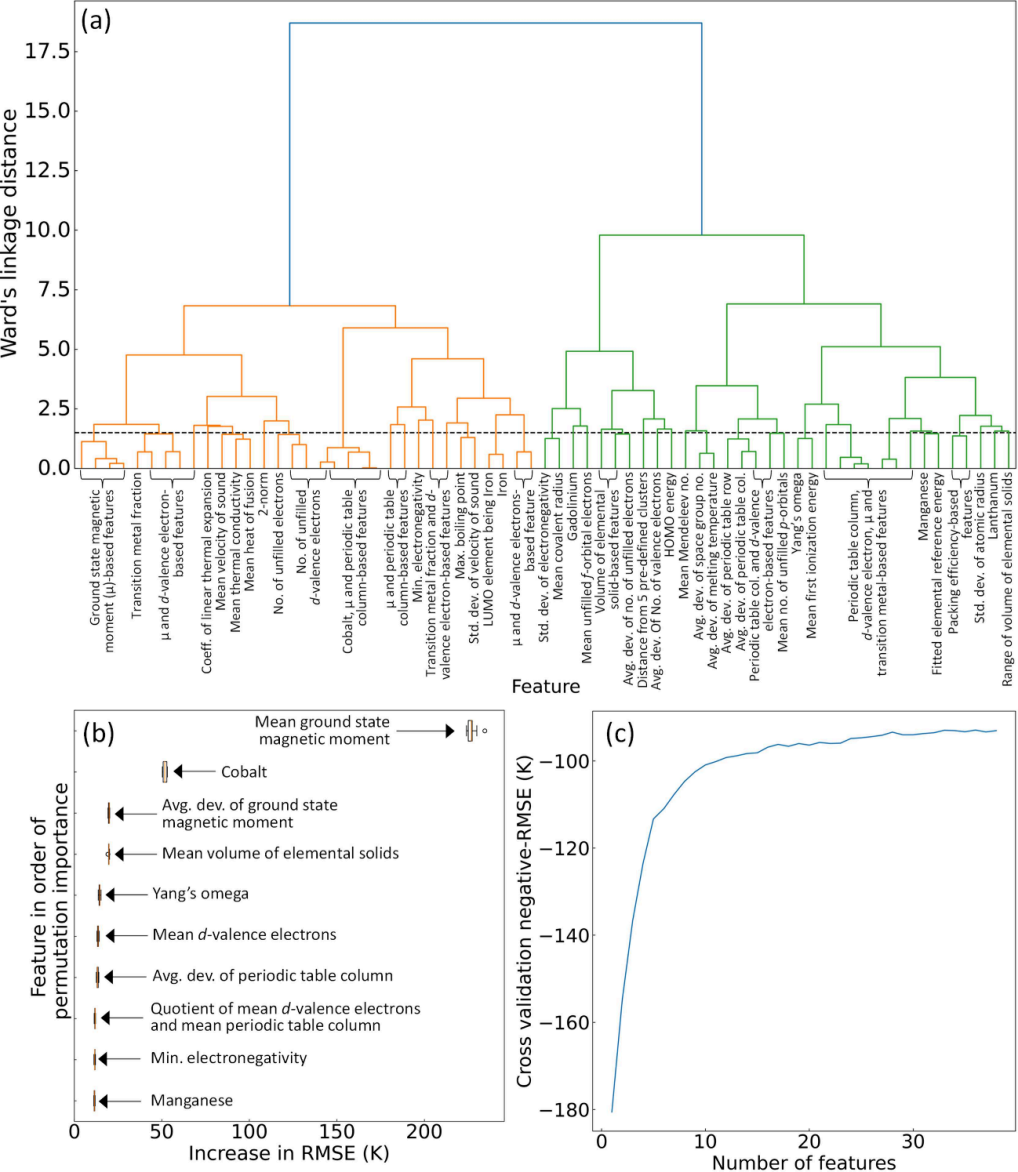
(a) Multicollinearity reduction - the dendrogram of the hierarchical agglomerative clustering using the remaining 67 features after performing the correlation analysis. The dashed horizontal line in black represents the distance threshold of 1.5 unit of Ward’s linkage distance. (b) The permutation feature-importance box plot for the regression of predicted *T*_*c*_ values. (c) The 10-fold recursive feature elimination result using the negative-RMSE as the performance metric.

To mitigate the effects of multicollinearity in the data set, features with a correlation coefficient of 0.8 or higher were systematically removed, resulting in a reduced subset of 67 features. The next remediation of multicollinearity effects involved employing a hierarchical cluster analysis, using Spearman rank-order correlation with a Ward’s linkage distance threshold of 1.5 units. This led to the retention of 38 features, as only one feature from each cluster was chosen. The optimal distance threshold was determined using the Elbow method with a step-size of 0.5 units. The corresponding dendrogram in Figure 7 (a) depicts the hierarchical agglomerative clustering of features with respect to Ward’s linkage distance, whereby clusters form as one ascends the dendrogram, while the results of the 10-fold permutation feature-importance analysis are shown in Figure 7 (b).

Permutation feature importance is quantified as the reduction in a model performance when a single feature used in the construction of the model is randomly shuffled. This process disrupts the association between the feature and the target, making the reduction in model performance indicative of the reliance of the model on that particular feature. The 10-fold feature permutation analysis suggests that the most important feature is the mean ground-state magnetic moment of elemental solids for atoms within a given chemical composition, as estimated using Magpie data, followed by the presence of cobalt in the chemical composition and the mean *d*-valence electrons. These results are consistent with the scientific findings of the statistical analyses that were conducted independently.

The optimal subset of the remaining features was determined by eliminating further features through 10-fold recursive feature elimination, employing negative RMSE values as the performance metric; see Figure 7 (c) for the results. This process led to the identification of the final subset of 36 features. It is helpful to remember that these short-listed features have been chosen from an initial pool of ca. 800 original features as well as 56 engineered features (cf. Section 3.1.4), which demonstrates their highest relevance to the target variable without any prior knowledge of the scientific domain.

#### 3.1.6 Model Optimization and SHAP Analysis

**Figure 8:**
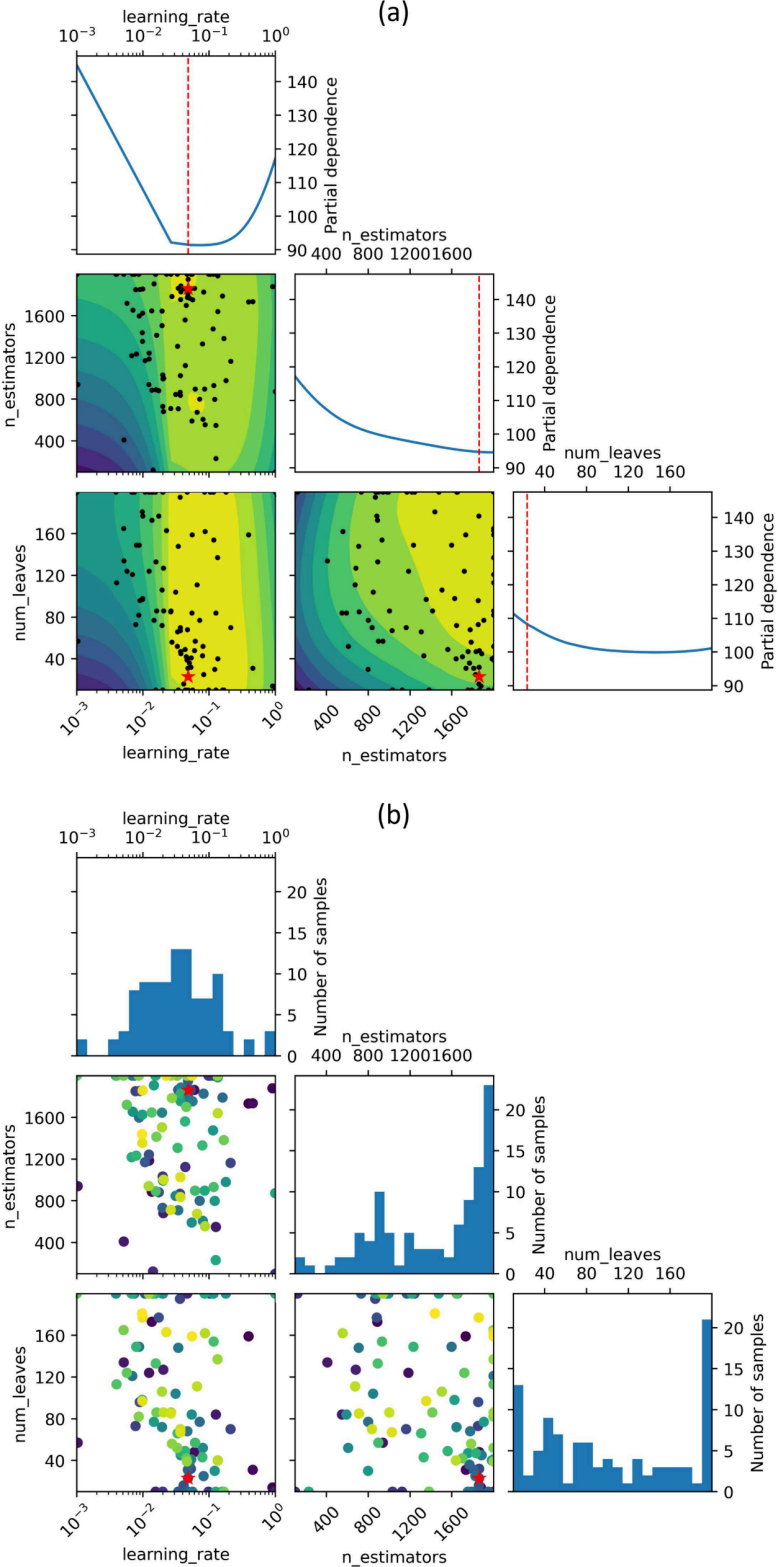
Bayesian optimization result of the final regression model using the training data, where (a) is the partial dependence plot and (b) is the evaluation plot. The red stars indicate the values of the hyperparameters that achieved the lowest value of the objective function. The approximate position of the objective minimum is indicated by the dashed vertical lines in red.

**Figure 9:**
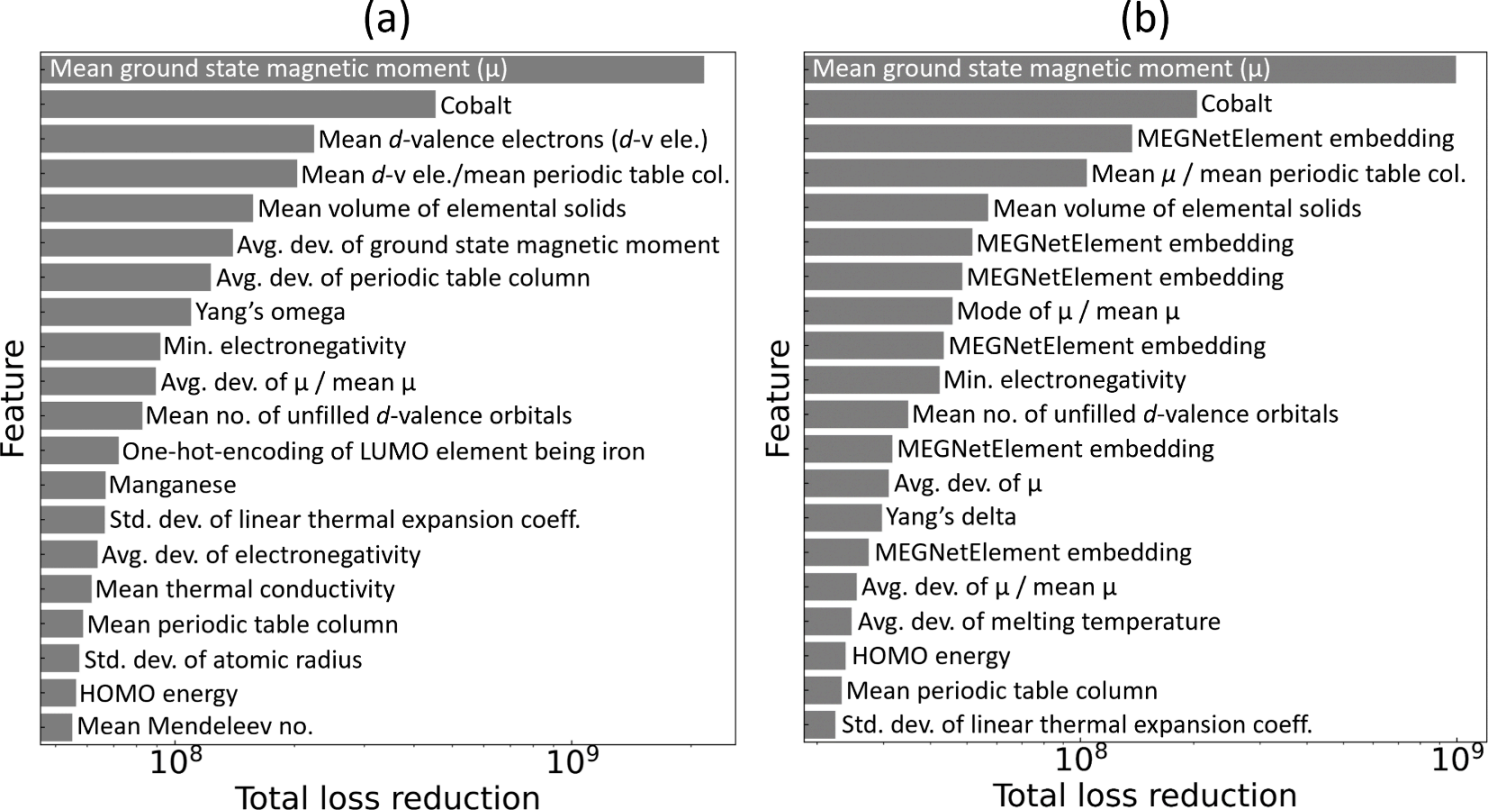
Feature relevance plot - top 20 features selected for the regression analysis of *T*_*c*_, where (a) is without and (b) is with MEGNet element embeddings, along with the realized total loss reduction (i.e., the relevance score).

A two-step optimization process was followed to determine the architecture of the final regression model. The hyperparameters of the model were optimized using a combination of grid search and Bayesian optimization using Gaussian processes. An initial hyperparameter tuning process was performed by scanning the hyperparameter space using the grid-search method. This subsequently identified the region in which Bayesian optimization was to be applied. Such an optimization strategy proves to be particularly effective for an objective function: (i) that has no closed form; (ii) that is expensive to evaluate; and (iii) whose evaluations yield noisy responses. The partial dependence and evaluation plots derived from the Bayesian optimization results are presented in Figure 8, while the total loss reduction (i.e., the feature-relevance ranking) that was realized by the top 20 features in the Bayesian-optimized model is illustrated in Figure 9.

**Figure 10:**
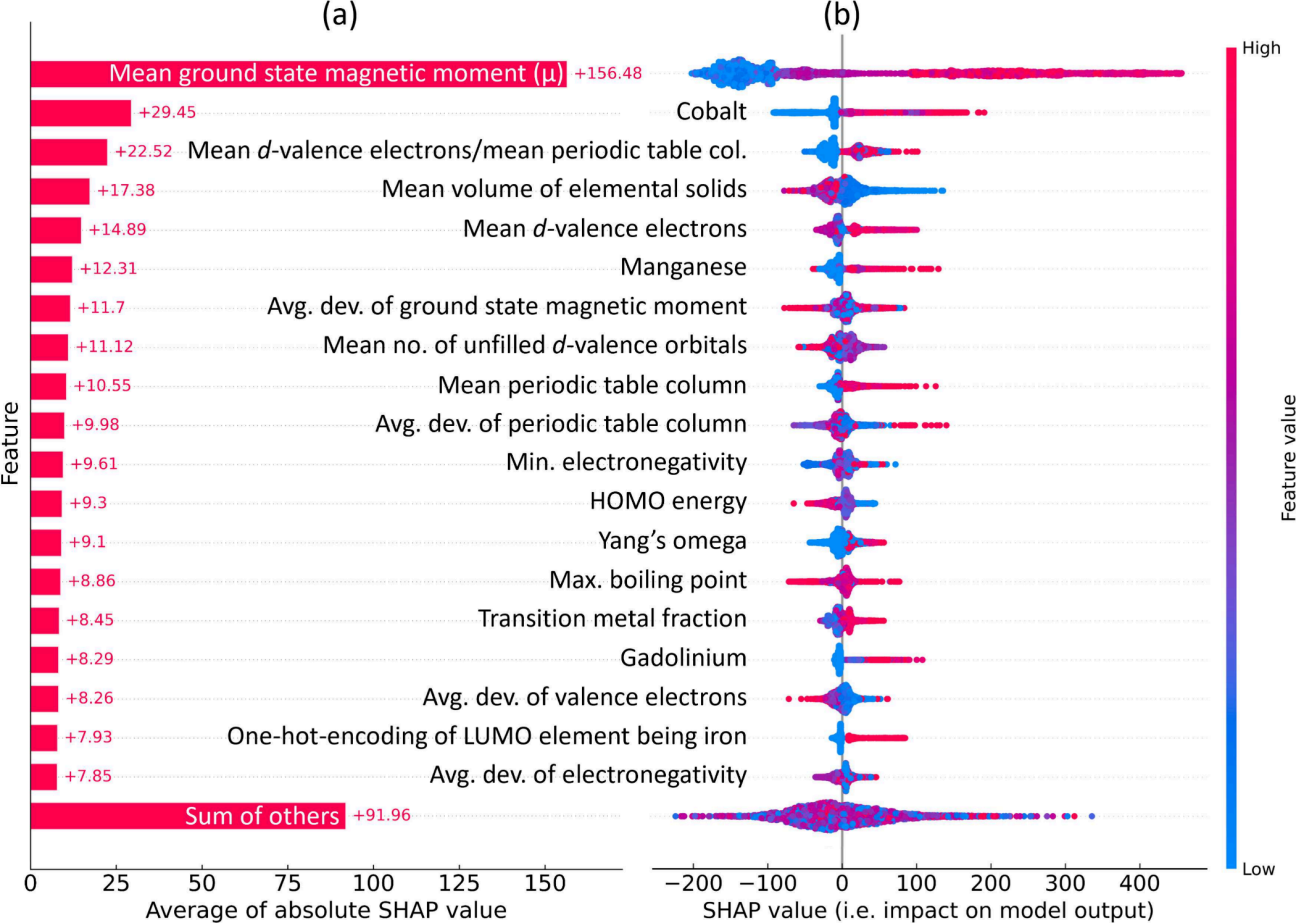
Results based on the SHAP framework: (a) the average contribution (i.e., the mean absolute SHAP value) of the ten features that are identified as having the greatest contributions to the model output. A positive SHAP value indicates a positive contribution to the regression of *T*_*c*_. (b) The beeswarm plot illustrates the impact of these features on the model output by plotting each instance as a single data point together with the SHAP value on the *x*-axis, where the *y*-axis is consistent with (a). The color scheme corresponds to the original feature value and the broadening shows the density of instances (cf. density plot).

An independent feature analysis was conducted using the SHapley Additive exPlanations (SHAP) framework,^59^ which is a game theoretic approach to explain the output of an ML model. Figure 10 (a) displays the plot of average contributions (i.e., the mean absolute SHAP value) of the ten features that have been identified as having the most significant contributions to the model output. The accompanying beeswarm plot in Figure 10 (b) illustrates the impact of these features on the model output by plotting each instance as a single data point together with the SHAP value on the *x*-axis. These findings align with the features that were identified by the GBFS workflow (see Figure 9), providing additional validation for the effectiveness of our modeling approach. Once again, we observe that the mean ground-state magnetic moment of elemental solids for atoms within a given chemical composition, the presence of cobalt, the mean *d*-valence electrons, and the mean volume of elemental solid, are among those identified as having the most significant contributions to the model prediction of *T*_*c*_ values. A substantial overlap is evident between the two sets of results.

### 3.2 Blind Test

The results discussed thus far present predictions of *T*_*c*_ values against literature values (cf. Figure 3), with promising statistical figures-of-merit. Nonetheless, it is important to validate these results by considering how these predictions fare across a diverse range of chemical materials rather than simply demonstrating their collective statistical quality in an anonymized form. Therefore, a comparative analysis was conducted against Data set 2, i.e., experimental measurements from the inorganic materials database, AtomWork, which contains ca. 16,000 chemical compositions with *T*_*c*_ values, of which ca. 6,600 are unique compositions. We applied our ML model to chemical compositions that were previously unseen by the model. The predictions of *T*_*c*_ values were subsequently compared against the experimental measurements, with 90 examples being summarized in Table 2. These examples were randomly chosen from instances where the predicted *T*_*c*_ value exceeded room temperature; more specifically, when *T*_*c*_ ≳ 600 K. This selection criterion is important, as materials exhibiting such *T*_*c*_ values are capable of maintaining thermally stable magnetic states or magnetization for functional applications.

**Table 2:**
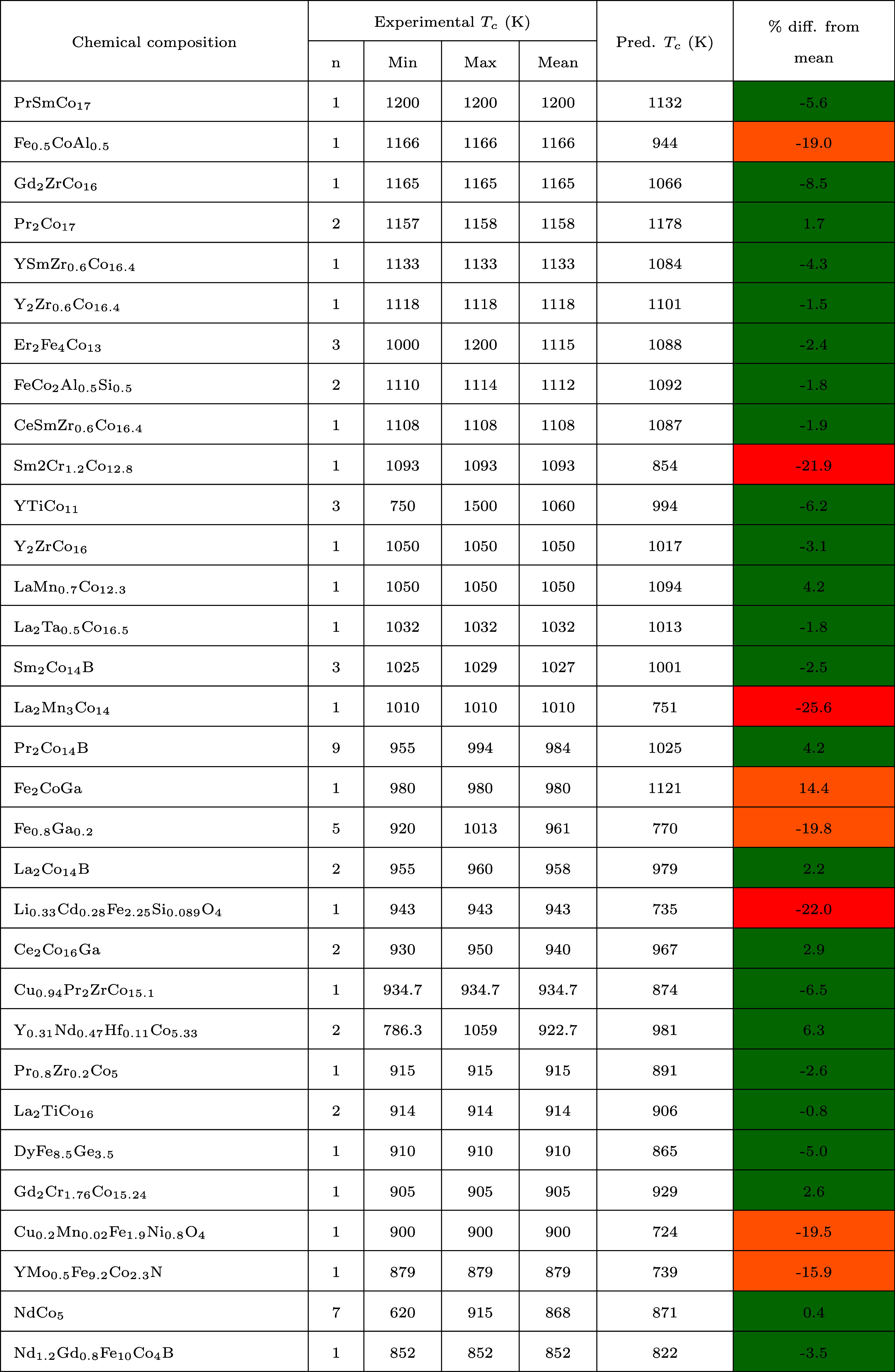

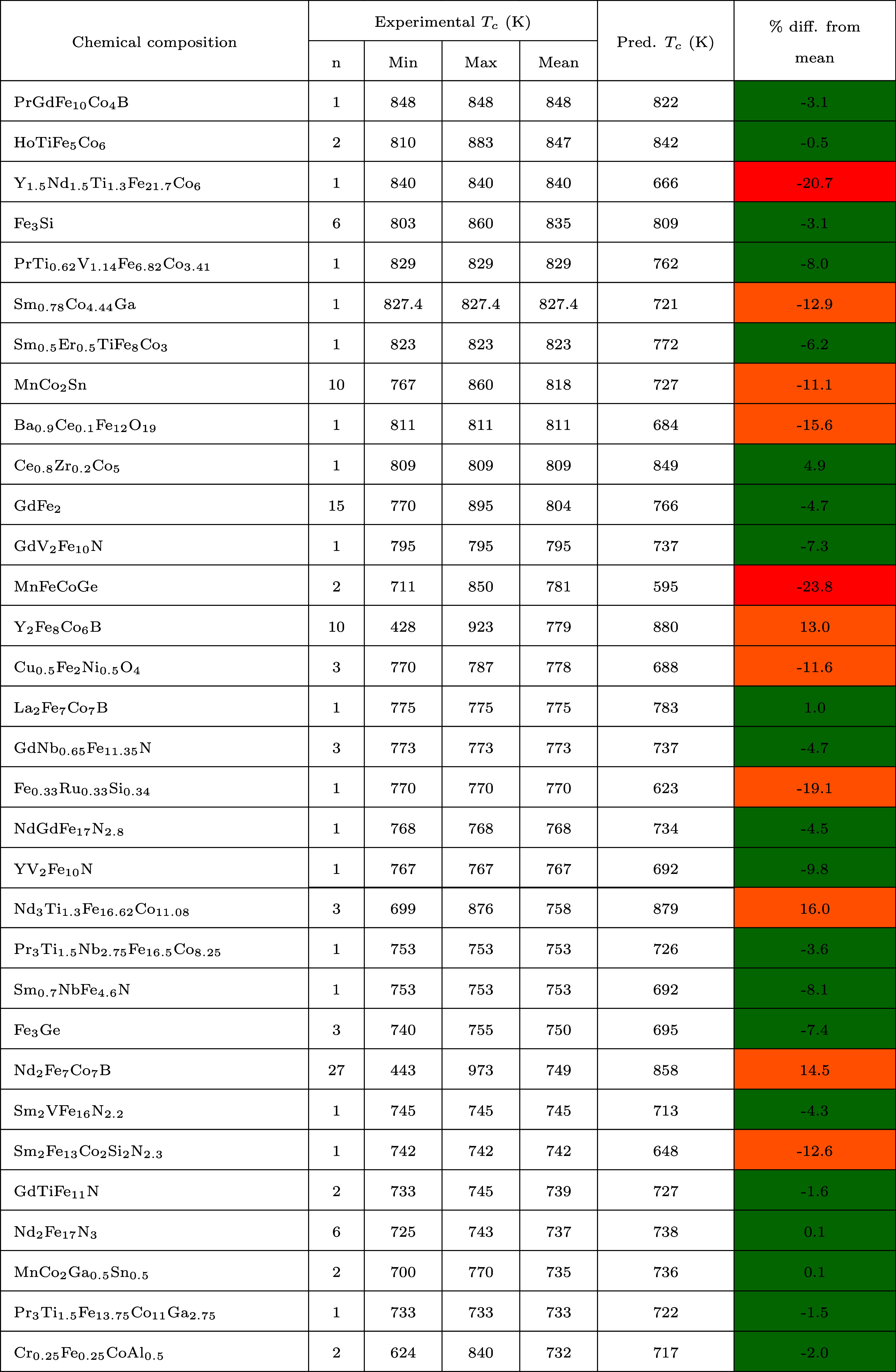

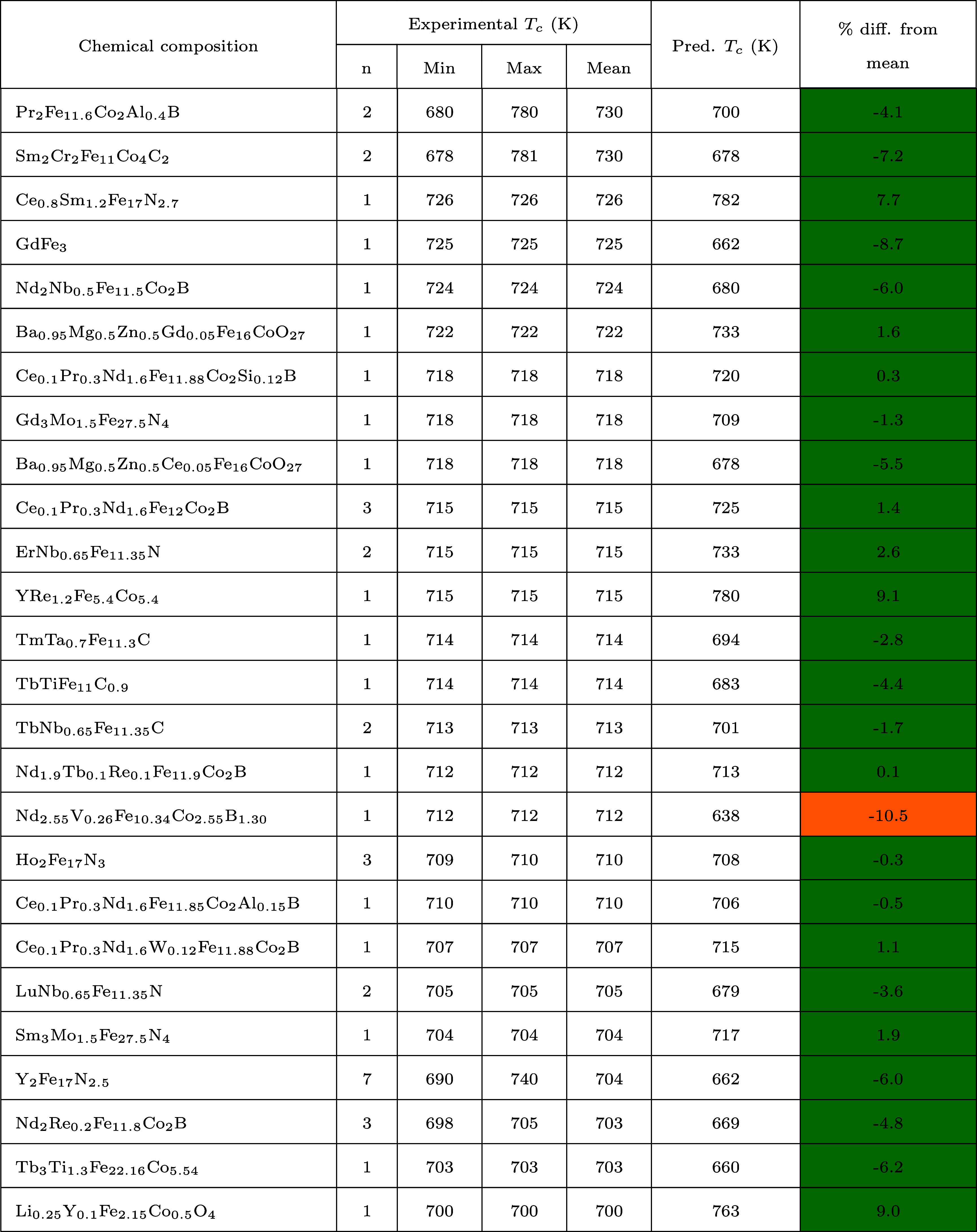
Examples of Chemical Composition (Model Input) and the Corresponding Prediction of *T*_*c*_ Values (Model Output), along with the Percentage Difference from the Mean Experimental *T*_*c*_ Values (Ground Truth)^a^

a The results are sorted by the magnitude of mean *T*_*c*_ values in descending order, where *n* is the number of experimental values sampled for each chemical composition; min and max represent their range; mean is their corresponding descriptive statistic. The absolute percentage differences between the predicted values and the experimental measurements are color-coded in green (0–10%), amber (10–20%), or red (>20%).

The aforementioned tendency of our model to underestimate experimental *T*_*c*_ values is apparent, with 64 out of 90 chemical compositions exhibiting a negative percentage difference from the mean *T*_*c*_ values. This underestimation becomes more evident at higher *T*_*c*_ values, as is depicted by the line of best fit shown in Figure 3 (a). We attribute this anomalous pattern to the scarcity of data related to ferromagnetic materials with high *T*_*c*_ values within the training set, making it challenging for ML models to learn effectively within this temperature range. We anticipate that the availability of a greater number of data points in these temperature ranges would enhance the efficacy of our ML models.

Notwithstanding this modest negative bias in our ML model, it predicts *T*_*c*_ values well on an absolute scale. Thereby, the average absolute percentage difference from the mean is ca. 6.7%. Some of the lowest absolute percentage differences were observed for chemical compounds such as MnCo_2_Ga_0.5_Sn_0.5_, Nd_1.9_Tb_0.1_Re_0.1_Fe_11.9_Co_2_B, and Nd_2_Fe_17_N_3_. Meanwhile, some of the highest absolute percentage differences were noted for chemical compounds such as La_2_Mn_3_Co_14_, MnFeCoGe, Li_0.33_Cd_0.28_Fe_2.25_Si_0.089_O_4_, and Sm_2_Cr_1.2_Co_12.8_, whose absolute percentage differences from the mean exceeds 20%. The absolute percentage difference of each predicted *T*_*c*_ value from its experimental measurement is color-coded according to the classifications: green (0–10%), amber (10–20%) or red (>20%). We observe that materials rich in cobalt and iron exhibit the highest *T*_*c*_ values. Among the 90 examples summarized in Table 2, iron and cobalt are present in the composition over 60 times. Other noteworthy chemical compositions include the elements, neodymium, samarium and manganese. The majority of the chemical compositions involve a combination of these chemical elements, as well as their oxide forms.

### 3.3 Predictive Capabilities for ML-Model Applications: Two Case Studies

We now explore potential applications of our ML models beyond benchmarking against state-of-the-art reports. Specifically, we aim to (i) predict Curie temperatures of rare-earth intermetallic compounds, and (ii) generate magnetic phase diagrams of chemical compounds, particularly those in a binary system, where *T*_*c*_ values are computed as a function of elemental composition. We assess how well the models perform when given the task to make out-of-distribution predictions. Here, out-of-distribution predictions are defined as model predictions that are made within a chemical space that is either under-represented or not represented by the data in the training set. Therefore, we are challenging our ML model to explore unfamiliar material spaces by extrapolating from patterns which pertain to chemical-property relationships that they have learned during the model training stage. This exploration will provide a clearer understanding of the predictive capabilities and suitability of our model for more practical applications.

**Figure 11:**
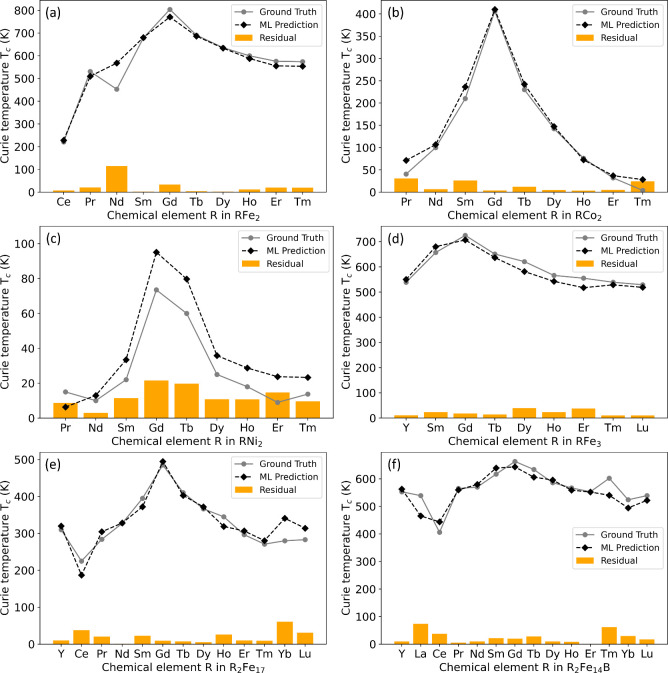
ML-based predictions of *T*_*c*_ values for unseen (a)–(c) RX_2_ and (d)–(f) R-Fe-based chemical compounds (black diamonds) against median experimental measurements that were obtained from *T*_*c*_ values in the literature (gray circles). The bar chart illustrates the residuals (orange bars), defined as the absolute discrepancies between the predicted values and the experimental measurements. Data from refs (1 and 60).

Figure 11 depicts the predicted *T*_*c*_ values (as black diamonds) of rare-earth intermetallic compounds against experimental measurements (i.e., the ground truth which is given as gray circles). Figures 11 (a) to (c) refer to unseen RX_2_ compounds (X = Fe, Co, Ni), where R represents a rare-earth element, while Figures 11 (d) to (f) correspond to more intricate R-Fe-based intermetallic compounds. The *x*-axis in each graph illustrates the chemical composition and the specific rare-earth elements considered in the prediction, with the ground truth being derived from refs (1 and 60). We emphasize that this prediction exercise extends the blind test, whereby model predictions are benchmarked against well-established data on known rare-earth-based compounds. Although these compositions are unseen by the model, they are familiar to the scientific community; therefore, this exercise is not about discovering new materials but rather about validating the predictive accuracy of our model.

Figure 11 shows that the majority of the *T*_*c*_ predictions made by our ML model for these unseen chemical compounds align well with the experimental measurements. However, a few notable deviations are evident. For instance, the predicted magnetic ordering temperature for NdFe_2_ is ca. 568 K, while the median experimental measurement is 453 K, as evidenced by the large discrepancy shown in Figure 11 (a). A closer inspection of the data set reveals that there are three independently measured experimental *T*_*c*_ values for this particular compound, which are quite disparate to each other: (i) 328 K, (ii) 453 K, and (iii) 578 K. Considering this, our prediction appears reasonable.

Furthermore, Figure 11 displays the results in order of ascending rare-earth atomic number. This revealed a discernible trend in these rare-earth intermetallic compounds: the incorporation of Gadolinium (Gd) into the chemical composition yields the highest *T*_*c*_ values in all cases. This pattern is evidenced by the prediction of our model, which aligns with experimental data, demonstrating a clear peak when R is Gd for the chemical material classes considered herein. This trend stands to reason given that Gd lies in the middle of the lanthanide series, whose elements are mostly stable in their R^3+^ electronic configuration. Thereby, the electronic configuration of Gd^3+^ ions is [Xe]4*f*^7^, 5*d*^0^, 6*s*^0^, i.e., all its *f* orbitals carry an unpaired electron which will maximize its magnetic moment. The peak *T*_*c*_ value for Gd will fall off fairly symmetrically as a function of an increasing or decreasing atomic number for R from that of Gd, since the number of unpaired electrons will decrease as the rare-earths extend to the lower and upper ends of the lanthanide series.

**Figure 12:**
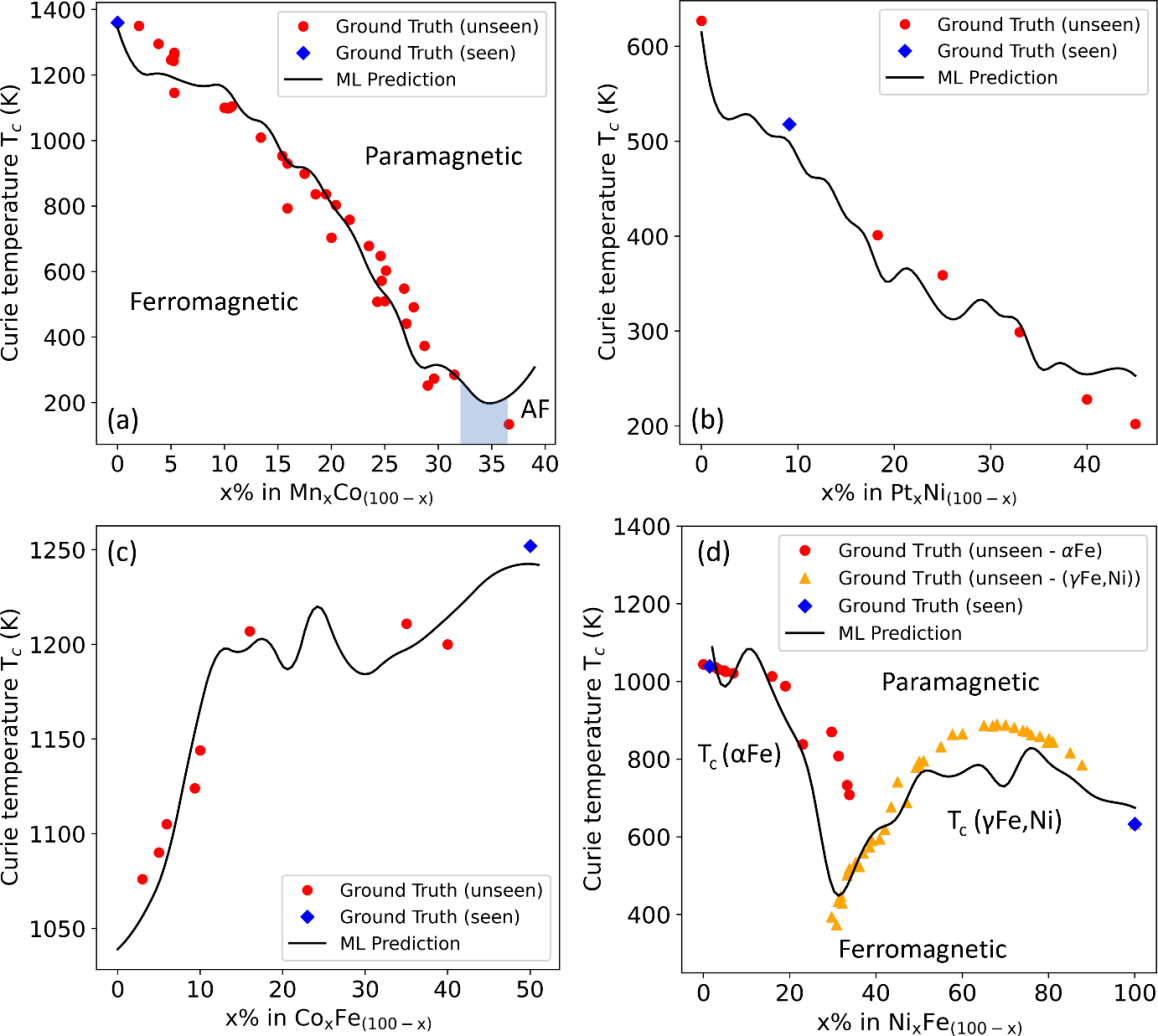
ML-generated magnetic phase diagrams - prediction of *T*_*c*_ as a function of elemental composition for (a) Mn–Co, (b) Pt–Ni, (c) Co–Fe, and (d) Ni–Fe binary systems. The results are compared against seen (diamonds) and unseen (circles and triangles) experimental measurements of *T*_*c*_ values from the literature. The shaded blue region in (a) depicts the approximate phase transition between the ferromagnetic and antiferromagnetic (AF) phases. The two types of unseen experimental measurements in (d) represent the two different Curie temperature profiles in the Ni–Fe binary system. Data from refs (27, 30, and 60−66).

Figure 12 illustrates the ML-generated phase diagrams for (a) manganese–cobalt (Mn–Co), (b) platinum–nickel (Pt–Ni), (c) cobalt–iron (Co–Fe), and (d) nickel–iron (Ni–Fe) binary systems; i.e., the prediction of *T*_*c*_ values as a function of an elemental composition for a given class of materials, with the ground truth being derived from refs (27, 30, and 61−66). The black line represents the ML predictions. Experimental measurements, unseen during the training of our ML model, are depicted by red circles and orange triangles, while those seen by the models are represented by blue diamonds. Specifically, the ML model was trained on a comprehensive range of chemical compositions that is available in the data set. However, compositions related to the given binary systems were excluded from the training set, with the exception of those represented by the blue diamonds. These exceptions indicate specific compositions within these binary systems that were intentionally included during the training phase. This methodological choice was adopted to demonstrate the model’s capability to make accurate out-of-distribution predictions, even with minimal or no data on the particular binary system under examination. Overall, we observe a strong correspondence between the predictions and the experimental measurements. The models demonstrate their ability to generate out-of-distribution predictions with a high level of accuracy. Figure 12 (d) further showcases the model’s proficiency in generating accurate out-of-distribution predictions, even in the presence of a transition between two phases in the Ni–Fe system, as indicated by the unseen experimental measurements marked in circles and triangles.

We now turn our attention to specific details within certain magnetic phase diagrams. The Mn–Co system has undergone extensive review by Ishida et al.^61^ and Men’shikov et al.^62^ Those studies elucidated the formation of disordered alloys and their crystal structures across a range of chemical compositions. The magnetic phase diagram of the Mn–Co binary system referred to in Figure 12 (a) encompasses regions of ferromagnetic and antiferromagnetic long-range order, along with superparamagnetic and superantiferromagnetic states. The latter states involve mixtures of ferromagnetic and antiferromagnetic clusters with the paramagnetic phase, respectively. Detailed magnetic phase diagrams can be referred to in the cited studies. Our discussion primarily focuses on the ferromagnetic state (from 0 to ca. 25–30 at % Mn) and neglects the antiferromagnetic state (above ca. 35 at % Mn) and the mixture states (in between the ferromagnetic and antiferromagnetic states), since the scope of this study is on ferromagnetic materials; besides, the experimental points for the mixture states are acknowledged to be less accurate, despite their established existence.

The profile of the magnetic ordering temperature of the Mn–Co system is known to monotonically decrease as a function of the Mn content from 0 to 30 at % Mn, before monotonically increasing above ca. 35 at % Mn. Our ML-generated magnetic phase diagram effectively reproduces these experimentally observed patterns, even though our model was trained exclusively on chemical composition-based features, with only elemental Co being seen by the model among the experimental data that pertains to this binary system. Surprisingly, the inversion point at the critical temperature at ca. 35 at % Mn is captured, despite corresponding to the antiferromagnetic region. The mean magnetic moment  within the ferromagnetic states decreases with an increase in Mn concentration relative to Co concentration (i.e., ). This signifies that in the Mn–Co alloy system, μ̅ diminishes due to the antiferromagnetic positioning of Mn atoms in relation to Co atoms. Specifically, the local magnetic moment (μ) of Mn atoms ranges from ca. 25 μ_*B*_ to 29 μ_*B*_ within the concentration range of 0 to ca. 20 at % Mn. The value of μ̅ continues to decrease beyond ca. 20 at % Mn due to a reduction in μ of Co atoms, whose values are influenced by Mn atoms through exchange interactions. The proportion of ferromagnetically interacting Co–Co pairs decreases until it reaches zero at a concentration of ca. 27 at % Mn, while the proportion of antiferromagnetically interacting Mn–Co and Mn–Mn pairs increases with a higher concentration of Mn. Therefore, the Mn–Co alloy system can be characterized as a typical Ising magnet, with antiferromagnetic interactions prevailing above a concentration of about 27 at % Mn. This observation aligns with the results obtained from our GBFS workflow, where the most important feature is identified as the mean magnetic moment of elemental solids for atoms within a given chemical composition, as one might expect.

Another magnetic phase diagram that warrants detailed discussion is the Ni–Fe alloy system (cf. Figure 12 (d)). In contrast to the Mn–Co alloy, the Ni–Fe alloy exhibits ferromagnetic states throughout its entire compositional range. The equilibrium phases of the Ni–Fe system encompass the: (i) liquid phase, (ii) body-centered cubic, high-temperature (δFe) solid solution, (iii) face-centered cubic (γFe,Ni) solid solution, (iv) body-centered cubic, low-temperature (αFe) solid solution, and (v) intermetallic compound of the form FeNi_3_.^63−66^ Our primary focus is on examining the *T*_*c*_ profile of the Ni–Fe alloy system associated with the Fe-rich (αFe) solid solution and (γFe,Ni) solid solution, as well as exploring the change in the *T*_*c*_ profile that corresponds to the two intermetallic phases.

Determining the phase boundaries in magnetic materials is challenging and their identification generally necessitates the use of advanced experimental materials-characterization methods such as powder X-ray diffraction and scanning transmission electron microscopy. Our ML-based magnetic phase diagram shown in Figure 12 (d) depicts a decreasing trend in *T*_*c*_ values as the Ni concentration increases from 0 to ca. 35 at % Ni. Beyond this point, an increasing trend is observed, closely following the experimental measurements with a peak *T_c_* of ca. 885 K at ca. 67 at % Ni, while the ML prediction shows a peak *T_c_* of ca. 830 K at ca. 76 at % Ni. Further increases in Ni concentration lead to a subsequent decline in the *T*_*c*_ value until a concentration of 100 at % Ni has been reached. It should be noted that an additional ferromagnetic phase involving the FeNi_3_ intermetallic compound is reported in the literature within the Ni concentration range of approximately 60 to 85 at % Ni. However, the magnetic ordering temperature of FeNi_3_ is not considered in this analysis, so we would not expect our ML model to identify such a phase. Our *T*_*c*_ predictions otherwise agree well with the experimentally determined magnetic phase diagram for Ni–Fe binary system that was produced by Swartzendruber et al.^64^

We note that our model for the Ni–Fe binary system successfully interpolates the chemical relationships derived from the training to make accurate out-of-distribution predictions, despite having encountered only two experimental measurements among those in this binary system during the training process; albeit, the training process is helped by the fact that these two experimental values lie at either end of the limiting compositional range of this binary system such that they give the model anchoring points with high statistical leverage. Our model identifies both the minimum and maximum *T*_*c*_ values, in addition to mirroring the experimental delineation of the *T*_*c*_ profiles corresponding to (αFe) and (γFe,Ni). Such predictions were not anticipated without structural information, especially considering the scarcity of experimental data points seen by the model across this compositional range. However, as discussed earlier, the mean magnetic moment of elemental solids appears to have played a crucial role in facilitating this prediction. In the ferromagnetic Ni–Fe system, μ̅ measured in the (αFe) phase ranges from 2.00 μ_*B*_ to 2.29 μ_*B*_, while in the (γFe,Ni) phase, it ranges from 0.61 μ_*B*_ to 1.93 μ_*B*_.^64^ This indicates a clear distinction in the range of μ̅ values between the two phases, from which the ML model appears to have extracted the information that is necessary to distinguish between the two intermetallic *T*_*c*_ phases. The utilization of magnetic measurements for the study of two-phase Fe–Ni alloys is described by Sucksmith et al.^67,67,68^ Lastly, it is crucial to acknowledge uncertainties associated with the examination of (αFe)/(γFe,Ni) phase boundaries. The transformation between these two phases can be influenced by the presence of material impurities. Additionally, environmental conditions during the varying experimental measurement processes will play an important role. For instance, applied pressure has been shown to lower the transformation temperature between the two phases (i.e., (γFe,Ni) → (αFe)).^64,69^ Therefore, discrepancies are expected among the experimental measurements reported in the literature.

## 4 Conclusions

This study has employed a machine-learning-based workflow for feature selection and statistical analysis to train predictive models for the Curie temperature (*T*_*c*_). Our feature-selection workflow integrates a distributed gradient boosting framework along with exploratory data and statistical analyses, as well as multicollinearity treatments. This pipeline identifies and selects a subset of features that are highly relevant to the target variable or class within a complex feature space, ensuring minimal feature redundancy and maximal relevance to the target variable or classes. Subsequently, gradient boosting trees are trained with the selected features, which are derived solely from the chemical composition of a material.

In an analysis involving ca. 11,000 chemical compounds with ca. 6,200 unique chemical compositions, our Bayesian-optimized regression model that predicts *T*_*c*_ values achieved an *R*^2^ of 0.93, an MAE of 38.8 K, and an RMSE of 72.2 K on a test set that was obtained via random splitting. A 10-fold cross-validation of this model yielded an *R*^2^ of (0.92 ± 0.01), an MAE of (40.8 ± 1.9) K, and an RMSE of (80.0 ± 5.0) K. These results are superior to those of complex algorithms reported in the literature that predict *T*_*c*_ values, some of which use intricate feature descriptors. This demonstrates the efficacy of our modeling approach and emphasizes the importance of thorough feature analysis and judicious selection over merely complex modeling. Additionally, a blind test was conducted on chemical compositions with *T*_*c*_ values sourced from AtomWork (Data set 2), which were not included in the initial training set (from Data set 1). An analysis of a randomly selected subset of this data set, comprising 90 chemical compounds whose *T*_*c*_ ≳ 600 K, revealed that materials abundant in cobalt and iron exhibited the highest *T*_*c*_ values. Notable chemical compositions also included the elements, neodymium, samarium, and manganese. The majority of the chemical compositions involved a combination of these chemical elements, as well as their oxide forms.

Finally, we investigated applications of our ML models beyond benchmarking against state-of-the-art reports. This exploration involved: (i) predicting *T*_*c*_ values of rare-earth intermetallic compounds and (ii) generating magnetic phase diagrams of chemical compounds within a binary system. These findings illustrate that our ML models possess the ability to make accurate out-of-distribution predictions by extrapolating the chemical-property relationships learned from the materials database.

## Data and Software Availability

We have made available the data and the code for the feature selection, statistical analyses, multicollinearity reduction, recursive feature elimination and Bayesian optimization at https://github.com/Songyosk/CurieML.
